# Multi-omics resources for targeted agronomic improvement of pigmented rice

**DOI:** 10.1038/s43016-023-00742-9

**Published:** 2023-05-11

**Authors:** Khalid Sedeek, Andrea Zuccolo, Alice Fornasiero, Annika M. Weber, Krishnaveni Sanikommu, Sangeetha Sampathkumar, Luis F. Rivera, Haroon Butt, Saule Mussurova, Abdulrahman Alhabsi, Nurmansyah Nurmansyah, Elizabeth P. Ryan, Rod A. Wing, Magdy M. Mahfouz

**Affiliations:** 1 Laboratory for Genome Engineering and Synthetic Biology, Division of Biological Sciences, King Abdullah University of Science and Technology, Thuwal, Saudi Arabia; 2 Center for Desert Agriculture, Biological and Environmental Sciences and Engineering Division, King Abdullah University of Science and Technology, Thuwal, Saudi Arabia; 3 Crop Science Research Center, Sant’Anna School of Advanced Studies, Pisa, Italy; 4 Department of Environmental and Radiological Health Sciences, Colorado State University, Fort Collins, CO USA; 5 Department of Agronomy, Faculty of Agriculture, Universitas Gadjah Mada, Yogyakarta, Indonesia; 6 Arizona Genomics Institute, School of Plant Sciences, University of Arizona, Tucson, AZ USA; 7 International Rice Research Institute, Strategic Innovation, Los Baños, Philippines

**Keywords:** Plant sciences, Biotechnology

## Abstract

Pigmented rice (*Oryza sativa* L.) is a rich source of nutrients, but pigmented lines typically have long life cycles and limited productivity. Here we generated genome assemblies of 5 pigmented rice varieties and evaluated the genetic variation among 51 pigmented rice varieties by resequencing an additional 46 varieties. Phylogenetic analyses divided the pigmented varieties into four varietal groups: *Geng-japonica*, *Xian-indica*, *circum-*Aus and *circum-*Basmati. Metabolomics and ionomics profiling revealed that black rice varieties are rich in aromatic secondary metabolites. We established a regeneration and transformation system and used CRISPR–Cas9 to knock out three flowering time repressors (*Hd2*, *Hd4* and *Hd5*) in the black Indonesian rice Cempo Ireng, resulting in an early maturing variety with shorter stature. Our study thus provides a multi-omics resource for understanding and improving Asian pigmented rice.

## Abstract (web summary)

Genome assemblies, genetic variations, and metabolome and metal ion profiles were generated for diverse pigmented Asian rice varieties. An early maturing, shorter-stature black rice variety was created using CRISPR–Cas9-mediated genome editing, providing insights for improving Asian pigmented rice.

## Main

Rice landraces show great genetic and phenotypic diversity. Many forms have pigmented pericarps due to anthocyanins and proanthocyanidins^1–4^. These metabolites, as well as other micronutrients, fatty acids, pre-biotics, antioxidants and fibre, account for the tremendous nutritional value of whole-grain pigmented rice^5^. Despite its nutritional value, most pigmented rice varieties have long life cycles (4 to 6 months) and suboptimal plant height^6,7^. We also lack a detailed analysis of the nutrient composition of these diverse rice varieties^8^.

Our first step in enabling efforts to improve pigmented rice was to provide comprehensive genomic information. Although the genomes of several different *japonica* and *indica* rice varieties have been assembled over the past decade, full genome sequences are available for only a handful of pigmented varieties^9,10^, limiting their usage in gene discovery and genome editing. Here we selected three black (Cempo Ireng, Pulut Hitam-2 and Balatinaw) and two red (Zag and Cempo Abang) rice varieties for whole-genome sequencing using the PacBio Sequel IIe platform. A total of 1.21–1.63 million high-quality circular consensus sequencing reads were obtained with a sequencing depth of 41.5–59.8-fold coverage (Supplementary Table 1). The reads were assembled using HiFi ASM^11^, and contigs were ordered, oriented and, if needed, scaffolded using the *Oryza sativa* Nipponbare reference genome (IRGSP RefSeq) as a guide. The five genome assemblies showed remarkable contiguity, as shown by the high N50 values and the small number of sequence gaps, and the genome sizes are comparable to the previously reported genome size of the IRGSP RefSeq^12^. The completeness of the five genome assemblies was assessed with the Benchmarking Universal Single-Copy Orthologue tool^13^, and this analysis was carried out using the gene dataset specific for Poales (poales_odb10.2020-08-05.tar.gz). This analysis identified 98.4% to 98.6% complete genes (Supplementary Table 2 and Supplementary Fig. 1), and these values were on par with or better than those obtained for the 16 platinum standard reference genomes recently assembled for *O. sativa* (95.7–98.6%)^14^.

AUGUSTUS software^15^ predicted more than 38,000 protein-coding genes per genome, consistent with previous rice genome annotations^16,17^ (Supplementary Table 1). The predicted protein-coding genes were unevenly distributed over the 12 chromosomes, with more genes on the chromosome arms/ends than towards the centromere (Supplementary Fig. 2). More than 70.6% of the genes had functional descriptions, including protein domains, motifs and homologues among the amino acid products annotated in IRGSP RefSeq. Gene ontology analyses assigned most of the genes to molecular functions (45–47%), followed by biological processes (38–41%) and functions associated with cellular components (14–15%) (Supplementary Data 1).

Insertions and deletions longer than 50 base pairs (bp) were identified by comparing the five assembled genomes with the IRGSP RefSeq using an ad hoc pipeline based on the long-read mapper NGMLR (https://github.com/philres/ngmlr) and the structural variant caller SVIM^18^. The number of genomic regions not shared between each of the five varieties and the IRGSP RefSeq ranged from 10,689 to 37,176 (Supplementary Table 3), probably reflecting the relative distance between the sequenced varieties. The overall content of transposable elements (TEs) and repetitive sequence of the five genome assemblies was 46.4–49.4% (Supplementary Data 2). The most represented TE class was the long terminal repeat retroelements, including the superfamily Ty3-gypsy.

**Fig. 1:**
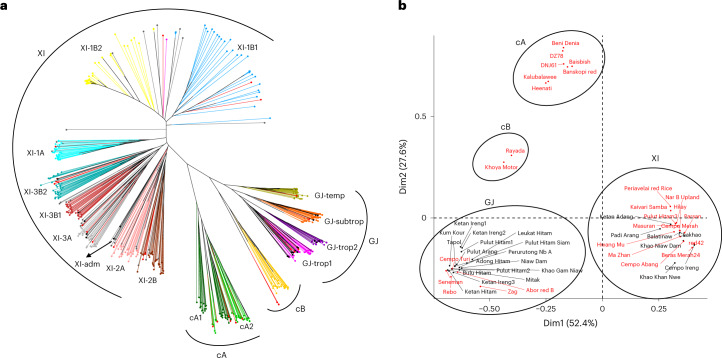
Population genomic analysis of pigmented rice. **a**, Neighbour-joining tree for *K* = 15 subpopulations and one admixture group. The phenogram shows the clustering of the 474 accessions selected from the 3K-RG dataset (clockwise: GJ-temperate (okra), GJ-subtropical (orange), GJ-tropical2 (dark magenta), GJ-tropical1 (magenta), cB (goldenrod), cA1 (dark green), cA2 (light green), XI-2B (brown), XI-2A (pink), XI-adm (dark grey), XI-3A (light grey), XI-3B1 (brick red), XI-3B2 (turquoise), XI-1A (cyan), XI-1B2 (yellow) and XI-1B1 (sky blue)) and the 51 pigmented varieties (the red and black branches represent red- or black-pigmented varieties, respectively). *O. sativa* subpopulations are defined as described by Zhou et al.^14^. **b**, Principal component analysis plot showing the clustering of the pigmented varieties into the four main groups of *O. sativa*. The analysis was performed on 51 pigmented varieties and 474 accessions selected from the 3K-RG dataset, but only the 51 pigmented varieties are shown. Red and black names represent red and black pigmented varieties, respectively.

To detect genetic variation among pigmented rice, we resequenced an additional 46 varieties (2× 150-bp paired-end reads) using the Illumina NovaSeq 6000 platform and generated an average of 42.35 million high-quality reads per variety (Supplementary Table 4). Resequencing depth ranged from a 10.37-fold to 60.56-fold genome coverage, and a 22.32-fold average mapping coverage was obtained by aligning the filtered reads to the IRGSP RefSeq (Supplementary Table 4). The Illumina reads along with in-silico-generated 2× 150-bp paired-end reads from the five high-quality genome assemblies were used for single nucleotide polymorphism (SNP) calling. Comparing the pigmented rice genomes with the IRGSP RefSeq identified 3,788,476 SNPs. The SNPs were used to assess the phylogenetic relationships of the pigmented varieties, taking advantage of the population structure and diversity revealed by the 3,000 Rice Genomes (3K-RG) project^19^. The 51 pigmented varieties were assigned to subpopulations on the basis of their genetic similarity to the 3K-RG dataset (Fig. 1a and Supplementary Tables 5 and 6). Most of the varieties were assigned to *Geng*-*japonica* (GJ) and *Xian*-*indica* (XI) groups (22 and 20 varieties, respectively). Seven varieties clustered into *circum*-Aus (cA), and two clustered into *circum*-Basmati (cB). The varieties assigned to GJ or XI clustered irrespective of the grain pigmentation. This result is consistent with the principal component analysis, which differentiated the varieties into the four major varietal groups (Fig. 1b).

**Fig. 2:**
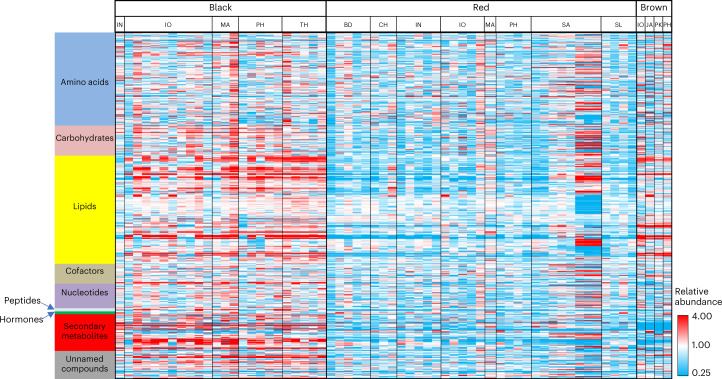
Global non-targeted metabolic screening of pigmented rice. Heat map visualization of differences in the median-scaled relative abundance of the identified metabolites in pigmented rice. Each cell represents a specific metabolite. Red cells indicate abundances higher than the median value, while blue cells indicate abundances below the median; white cells are abundances similar to the median. IN, India; IO, Indonesia; MA, Malaysia; PH, Philippines; TH, Thailand; BD, Bangladesh; CH, China; SA, Saudi Arabia; SL, Sri Lanka; JA, Japan; PK, Pakistan.

Our next step in enabling the improvement of pigmented rice was to comprehensively characterize the metabolites and elemental composition of these varieties; these data allow us (and other researchers aiming to improve these varieties) to identify varieties with superior nutrition for further improvement and to identify key traits to improve in selected varieties. To this end, we screened the metabolome profiles of 63 diverse pigmented Asian rice varieties to elucidate their composition. In total, 625 biochemicals were identified (Supplementary Data 3). About 60% of the compounds (375) significantly differed in abundance between black rice (BR) and red rice (RR), with the vast majority of these being higher in BR, especially secondary metabolites from the phenylpropanoid pathways and lipids (Fig. 2 and Supplementary Fig. 3). We identified 212 significantly different compounds between BR and brown rice (BrR) and 158 between BrR and RR. BR exhibited much higher levels of flavonoids from the proanthocyanin class, while RR had higher concentrations of proanthocyanidin pigments (Supplementary Fig. 3). The elevated production of other phenylpropanoid intermediates and chlorogenic acids were also associated with the BR group (Supplementary Fig. 4). Less expected were findings that lipid metabolism differed between the genotype groups, specifically that both BR and BrR contained similarly high levels of several classes of lipid catabolic products in comparison with RR, suggesting more active lipolysis in BR and BrR (Supplementary Fig. 5). In addition, a subset of the rice varieties was found to contain fagomine, an imino sugar alkaloid that has not been previously identified in rice. (Supplementary Data 3). This compound, first identified in buckwheat, has favourable bioactivities related to blood glucose management and insulin resistance^20,21^. Overall, our analysis reveals that BR is the most nutritious type of rice across a comprehensive suite of secondary metabolites, carbohydrates, amino acids, lipids, peptides and vitamins (Supplementary Fig. 3).

Moreover, we screened the metal ion profiles of the same rice varieties and identified and quantified 22 metal ions (Supplementary Data 4 and Supplementary Fig. 6). Essential microelements, such as Fe, Zn, Cu, Mn and Se, play key roles in numerous metabolic processes in the body. They are needed in trace amounts for proper human growth and development and are therefore considered potential candidates for crop biofortification to improve nutritional value and reduce the risk of deficiency-related diseases^22^. Fe and Zn deficiency is the most prevalent micronutrient deficiency, affecting more than two billion people globally, and is a major cause of early childhood mortality, mainly in developing countries^23^. Our analysis shows that dehusked, whole-grain pigmented rice, especially BR genotypes, are rich in these essential microelements. In particular, Cempo Ireng (the richest rice in Fe and the richest BR genotype in Zn) could provide the daily requirements of these essential elements.

We next used our metabolic and metal ion profiling data to identify several nutrient-rich varieties with higher levels of antioxidants and other healthy compounds and beneficial elements—namely, Pulut Arang, Pulut Hitam Siam, Cempo Ireng, Leukat Hitam, Pulut Hitam-2, Ketan Ireng-1 and Kum Kour. These can be considered candidate rice varieties for trait improvement. Cempo Ireng was the variety richest in Fe and vitamin B_2_ and was the BR variety with the highest Zn content. Despite its pest and disease resistance^7,24^, farmers are reluctant to cultivate Cempo Ireng due to its long life cycle (about five months) and long lax culm (up to 130 cm), making it prone to yield loss from lodging and bird attack. Flowering time (heading date in cereal crops) is one of the most important agronomic traits for rice cultivation and is controlled by several genetic and environmental factors. Three major photoperiodic flowering suppressors in the Early heading date 1 (Ehd1) pathway of rice are *Hd2/DTH7*, *Hd4/Ghd7* and *Hd5/Ghd8/DTH8* (refs. ^25–29^). These loci also affect plant height; therefore, we can improve flowering time and lodging resistance simultaneously.

Here we targeted the individual knockout of all three genes in Cempo Ireng using CRISPR–Cas9-mediated genome editing. To this end, we first established efficient regeneration and *Agrobacterium*–mediated transformation protocols for Cempo Ireng (Supplementary Sections 4 and 5). Although efficient methods for regeneration and transformation have been established for the *japonica* variety Nipponbare, the germplasm of other rice varieties (especially those used in agriculture) varies significantly in its response to callus induction, regeneration and transformation^30,31^. We next used this system to introduce CRISPR–Cas reagents into this variety (Supplementary Table 7). Fifteen homozygous T_1_ mutants (five for each target) and five wild-type plants were analysed for their heading date and other phenotypic traits. The mutant plants flowered and set seeds normally but significantly earlier than the wild-type control. The heading dates of *hd2*, *hd4* and *hd5* were decreased by about 27, 33 and 32 days, respectively (Supplementary Fig. 9), indicating the ability of CRISPR technology to accelerate the maturation cycle of BR. In addition, the mutant lines were 8–16 cm shorter than the wild type; however, this reduction was significant only for the *hd4* mutants.

This work provides important resources that give a clear roadmap for stakeholders (including crop bioengineers and breeders) to install desirable traits of value and to introduce pigmented rice into the food chain to improve human health and reduce the burden of malnutrition in developing and developed countries. However, whether these engineered traits can successfully co-exist with the traits of value in pigmented rice remains to be tested. Moreover, we identified several nutritious BR varieties worthy of further investigation as priorities for improvement by the targeted change of undesirable agronomic traits to enhance their overall productivity. However, more work is needed to establish Cempo Ireng and other pigmented rice varieties as superfoods. For example, the overall yield can be improved by targeting genes enhancing yield-related traits, such as *GS3*, *GW2* and *Gn1a*, thereby encouraging farmers/investors to cultivate large areas. Also, heavy-metal uptake can be reduced by avoiding cultivation in contaminated soil and using clean water resources, or using genome-editing technology to alter membrane channels to selectively take up beneficial but not toxic metals. Other technologies may help expedite the generation of pigmented rice with these traits of value, including speed breeding^32^. Although improving the productivity and shortening the life cycle of pigmented rice requires multiple steps, our work enables efforts to support human health via an improved diet that includes pigmented rice rich in micronutrients and vitamins.

## Methods

### PacBio sequencing

We selected three BR (Cempo Ireng, Pulut Hitam-2 and Balatinaw) and two RR (Cempo Abang and Zag) varieties for genome sequencing. Leaf tissue (~20 g) was used for genomic DNA extraction using the CTAB method^33^. The DNA was gently sheared into fragments (10–30 kbp) using Covaris g-TUBE, followed by bead purification with PB Beads (PacBio). The sequencing libraries were then constructed following the manufacturer’s protocol using the SMRTbell Express Template Prep kit v.2.0. Sequencing was performed using SMRT Cell 1M chemistry v.3.0 on a PacBio Sequel II system in circular consensus sequencing mode. The genome assemblies were carried out using HiFi Asm v.0.7 (ref. ^11^) with the default settings. Contigs from the primary assemblies were then mapped onto the *O. sativa* Nipponbare reference genome using the Mashmap tool^34^. The results were visualized as dot-plot comparisons, and the contigs were arranged into pseudomolecules. All reassembled genomes were compared with Nipponbare to search for structural variants using the ad hoc devised pipeline described by Zhou et al.^14^. Searching for TEs and repetitive sequences was carried out using RepeatMasker (http://www.repeatmasker.org/) run under the default parameters (except the qq option) and the rice TE library 7.0.0.liban described by Zhou et al.^14^. Gene prediction was carried out using the OmicsBox tool^35^, which relies on AUGUSTUS software^15^. The gene predictions were made by referencing the publicly available training set devised for *O. sativa*, along with extrinsic data, including 77,217 sequences and 67,138,695 paired-end RNA-seq sequences collected from four different tissues of *O. sativa*. The predictions were filtered using a 0.6 threshold for posterior probability, as provided by AUGUSTUS. Gene annotation was performed according to the best match for each predicted protein against the non-redundant National Center for Biotechnology Information protein database using Diamond BLASTp (v.0.9)^36^. A gene ontology analysis was performed using InterProScan v.5.39 with the default settings^37^. We sequenced another 46 pigmented rice varieties using the NovaSeq 6000 S1 Reagent Kit v.1.5 (Illumina) (Supplementary Section 1).

### Non-targeted global metabolic screening

We screened the metabolic profiles of 24 BR, 35 RR and 4 BrR varieties. The mature grains were ground in liquid nitrogen and lyophilized for 30 h, after which the samples were prepared by Metabolon Inc. Several recovery standards were added prior to the extraction process. The samples were extracted with 80% methanol under vigorous shaking for 2 min (Glen Mills GenoGrinder 2000). The resulting extract was divided into four fractions: two for analysis by reversed-phase ultra-performance liquid chromatography–tandem mass spectrometry (RP/UPLC–MS/MS) methods using positive ion mode electrospray ionization (ESI), one for analysis by RP/UPLC–MS/MS using negative ion mode ESI and one for analysis by HILIC/UPLC–MS/MS using negative ion mode ESI. All methods utilized a Waters ACQUITY UPLC and a Thermo Fisher Scientific Q-Exactive high-resolution/accurate mass spectrometer interfaced with a heated ESI (HESI-II) source and an Orbitrap mass analyser operated at 35,000 mass resolution. Each sample extract was dried and then reconstituted in solvents compatible with each of the four methods. Each reconstituted solvent contained a series of standards at fixed concentrations to ensure injection and chromatographic consistency. The MS analysis alternates between MS and data-dependent MS^n^ scans using dynamic exclusion. The scan range varies slightly between methods but covers approximately 70–1,000 *m*/*z*. Raw UPLC–MS/MS data were extracted and filtered to remove those representing system artefacts, misassignments, redundancy and background noise. Peaks and compounds were identified by comparison to library entries of the purified standards, and the peaks were quantified as area-under-the-curve detector ion counts; Welch’s two-sample *t*-test was used to analyse the data.

### Metal ion profiling

We quantified the metal ion content of the same 63 rice varieties. The grain powder was homogenized in water, and 4 mg per sample was mixed with 250 µl of nitric acid (16 M) and incubated for 2 h at 25 °C. Then, 25 µl of hydrochloric acid (12 M) was added, and the samples were centrifuged for 1 min at 1,500 *g*. The samples were heated at 90 °C for 1 h and then cooled before adding 100 µl of hydrogen peroxide (9.8 M). The samples were dried at 90 °C and were reconstituted with 1 ml of 0.5% nitric acid. In addition to the experimental samples, quality control samples including six blanks (diH_2_O), eight technical replicates made from pooled experimental samples and two samples of well-characterized pooled human plasma were included in the digestion and analysis. The samples were introduced into the inductively coupled plasma MS via an ESI Prep-Fast autosampler. The Thermo Fisher Scientific ICAP-RQ instrument was operated in positive ionization and used a KED cell to reduce polyatomic interference. Quantitation was performed using a multi-point external calibration curve (Mn, Co and Mg used a 16-point curve to accommodate this specific matrix), and internal standards were used to account for sample-specific suppression^38^.

### CRISPR–Cas9-targeted modification

We designed one single guide RNA to knock out Cempo Ireng *Hd2*, *Hd4* and *Hd5* genes and cloned it into the pRGEB32 vector under the Os*U3* promoter (Supplementary Table 8). All binary vectors were used for rice transformation by *Agrobacterium tumefaciens* strain EHA105. We genotyped the transformed plants by PCR using transfer-DNA-sequence-specific primers (Cas9-F7 and Nos-R7). The PCR amplicons encompassing the targeted region were cloned into a pJET vector (Thermo Fisher Scientific). We conducted Sanger sequencing for individual clones to determine the nature of sequence modification. We phenotyped the modified plants for heading date and yield-related traits (Supplementary Section 4).

### Reporting summary

Further information on research design is available in the Nature Portfolio Reporting Summary linked to this article.

## Supplementary information

Supplementary Information Supplementary Figs. 1–10, Tables 1–8, Methods and Results.

Reporting Summary

Supplementary Data 1 Functional annotation of the predicted genes.

Supplementary Data 2 Repetitive sequences in the genome of pigmented rice.

Supplementary Data 3 Metabolic profiling of pigmented rice.

Supplementary Data 4 Metal ion profiling of pigmented rice.

Supplementary Data 5 Correlation analysis between grain pigments and metal ion concentrations.

Supplementary Data 6 Unprocessed gel image and source data for Supplementary Fig. 9.

## Data Availability

The data supporting the findings of this study are included within the article and its Supplementary Information files. The raw genomics sequences and assemblies have been deposited to the National Center for Biotechnology Information under the BioProject accession code PRJNA942452. The MS metabolomic data have been deposited to the MetaboLights database with the identifier number MTBLS3320.

## Code availability

All custom code used in this study is available from the corresponding author upon request.
