# Supplementary material for: Multi-omics resources for targeted agronomic improvement of pigmented rice
Source: Nat Food. 2023 May 11;4(5):366–71. doi: 10.1038/s43016-023-00742-9 (PMC10208962; doi:10.1038/s43016-023-00742-9)
Supplement: Supplementary file 1 — Supplementary Figs. 1–10, Tables 1–8, Methods and Results. [file 43016_2023_742_MOESM1_ESM.pdf]

nature food

Brief Communication https://doi.org/10.1038/s43016-023-00742-9

Multi-omics resources for targeted agronomic improvement of pigmented rice

In the format provided by the authors and unedited

Supplementary Information

This file contains:

Supplementary S1

- Illumina genome resequencing.
- **Supplementary Fig. 1.** Dot plot comparing the genome assemblies of the five varieties and the reference Nipponbare genome.
- **Supplementary Fig. 2.** Genomic landscapes of the 12 chromosomes in the five genome assemblies of pigmented rice.
- **Supplementary Table 1.** Summary of whole-genome sequence assembly, annotation, gene prediction, and structural variants.
- **Supplementary Table 2.** BUSCO assessment of the five genome sequence assemblies.
- **Supplementary Table 3.** Structural variants (SVs) distribution in pigmented rice genome sequences.
- **Supplementary Table 4.** Resequencing of pigmented rice accessions.
- **Supplementary Table 5.** List of pigmented rice accessions and corresponding subpopulations.
- **Supplementary Table 6.** Rice accessions used for phylogenetic analysis.

Supplementary S2

- **Supplementary Fig. 3.** Comparison of the average metabolite abundance among pigmented rice varieties.
- **Supplementary Fig. 4.** Box plots of selected secondary metabolites differing significantly by the grain color variable.
- **Supplementary Fig. 5.** Box plots showing examples of lipid species differentially expressed in the different types of pigmented rice.

Supplementary S3

- **Supplementary Fig. 6.** Metal ion profiling of the pigmented rice.
- Relationship between grain pigments and metal ions.

Supplementary S4

- Embryogenic callus induction and regeneration
- Agrobacterium-mediated transformation.
- Genomic DNA extraction and genotyping.
- Phenotyping of CRISPR/Cas9-targeted Cempo Ireng.

Supplementary S5

- Establishment of a regeneration and transformation system for BR.

Supplementary S6

- Media Setup
- **Supplementary Fig. 7.** Main steps of the establishment of a regeneration protocol for Cempo Ireng.
- **Supplementary Fig. 8.** Different stages of the *Agrobacterium*-mediated transformation of Cempo Ireng.
- **Supplementary Fig. 9.** CRISPR/Cas9-induced knockout of three repressors of heading date in rice.
- **Supplementary Fig. 10.** Protein sequence alignment of the CRISPR mutants and the wild type (Cempo Ireng).
- **Supplementary Table 7.** Genotyping of the T0 and T1 edited plants.
- **Supplementary Table 8.** List of oligos.

Supplementary S1

Illumina genome resequencing. The CTAB method was used to extract high-quality genomic DNA from the two-week-old leaves of 46 pigmented rice varieties. The genomic DNA was physically sheared into fragments of 250–1000 bp, after which the libraries were constructed by targeting 350-bp inserts following standard Illumina protocol recommendations. The libraries were prepared by NovaSeq 6000 S1 Reagent Kit v1.5 (Illumina) and sequenced on the Illumina NovaSeq 6000 platform in 2×150-bp paired-end mode. Illumina read adapters from the 46 varieties were masked using Cutadapt v2.3 [1]. Trailing low-quality and masked bases were trimmed using Trimmomatic v0.38 [2]. Reads shorter than 50 bp after the trimming step were discarded. Synthetic Illumina reads (2×150 bp) were generated from the five varieties sequenced with PacBio CCS using the tool seqret included in the EMBOSS package (www.ebi.ac.uk) and added to the panel of 46 Illumina resequenced varieties.

Reads from the 51 varieties were aligned against the *O. sativa* Nipponbare (IRGSP RefSeq) reference genome using BWA-MEM (v0.7.17) [3], and then sorted using Samtools v1.8. [4]. The aligned reads were filtered using a minimum mapping quality of 30 ($-10 \log_{10}$ Probability). *FixMateInformation* from the Picard v2.17.16 tool suite (http://broadinstitute.github.io/picard/) was used to ensure all mate-pair information was in sync for each read pair. Read duplicates and artifacts were marked using Picard's tool *MarkDuplicates*. An alignment summary, insertion size metrics, and coverage metrics were collected for each sample. *HaplotypeCaller* (GATK v4.2.2.0) [5, 6] was used to call variants in the 51 varieties individually, using *GVCF* as the emitting reference confidence mode and setting a minimum base quality score of 30.

CombineGVCFs and *GenotypeGVCFs* (GATK v4.2.2.0) were used to perform a joint genotyping of the 51 varieties and an additional subset of 474 *O. sativa* accessions belonging to the 3K-RG project [7]. The 3K-RG accessions were evenly selected from the 15 subpopulations and the admixture subgroup of *O. sativa* identified by Zhou et al. [8], based on: i) a minimum coverage of 14× and ii) a maximum number of 32 varieties per subpopulation. The 16 Platinum Standard reference genomes [8] were included in the subset. The variant calling for the joint dataset was then filtered to only retain biallelic SNP sites and using the "core SNP set" filtering criterion described by Wang et al. [7]. Vcftools v0.1.17 [9] was used to discard any loci showing missing genotypes from the joint genotype dataset. Plink v5.3 [10] was used to build a square, symmetric matrix of the IBS distances for all pairs of individuals. Function *prcomp* in R v3.6.0 (https://CRAN.R-project.org/) was used to perform the PCA using the distance matrix as input, and the results were plotted using the R package *factoextra* (https://CRAN.R-project.org/package=factoextra). The distance matrix was converted into the PHYLIP format (Retief. 2000) and used as an input in DARwin 6.0.21 (https://darwin.cirad.fr/) to generate an unweighted NJ phenogram.

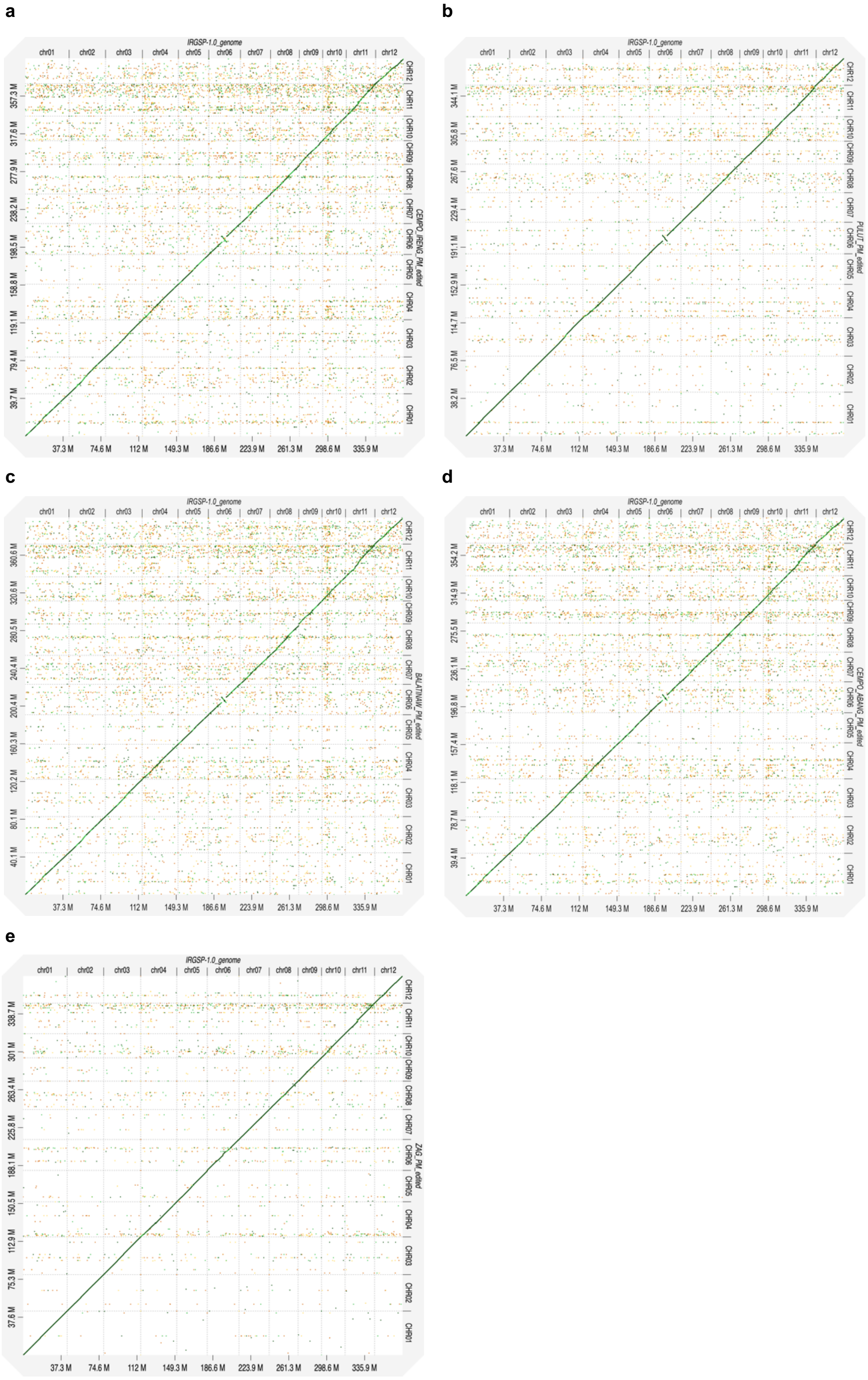

Supplementary Fig. 1. Dot plot comparing the genome assemblies of the five varieties and the reference Nipponbare genome. a) Cempo Ireng, b), Pulut Hitam-2, c) Balatinaw, d) Cempo Abang, and e) Zag genome assemblies.

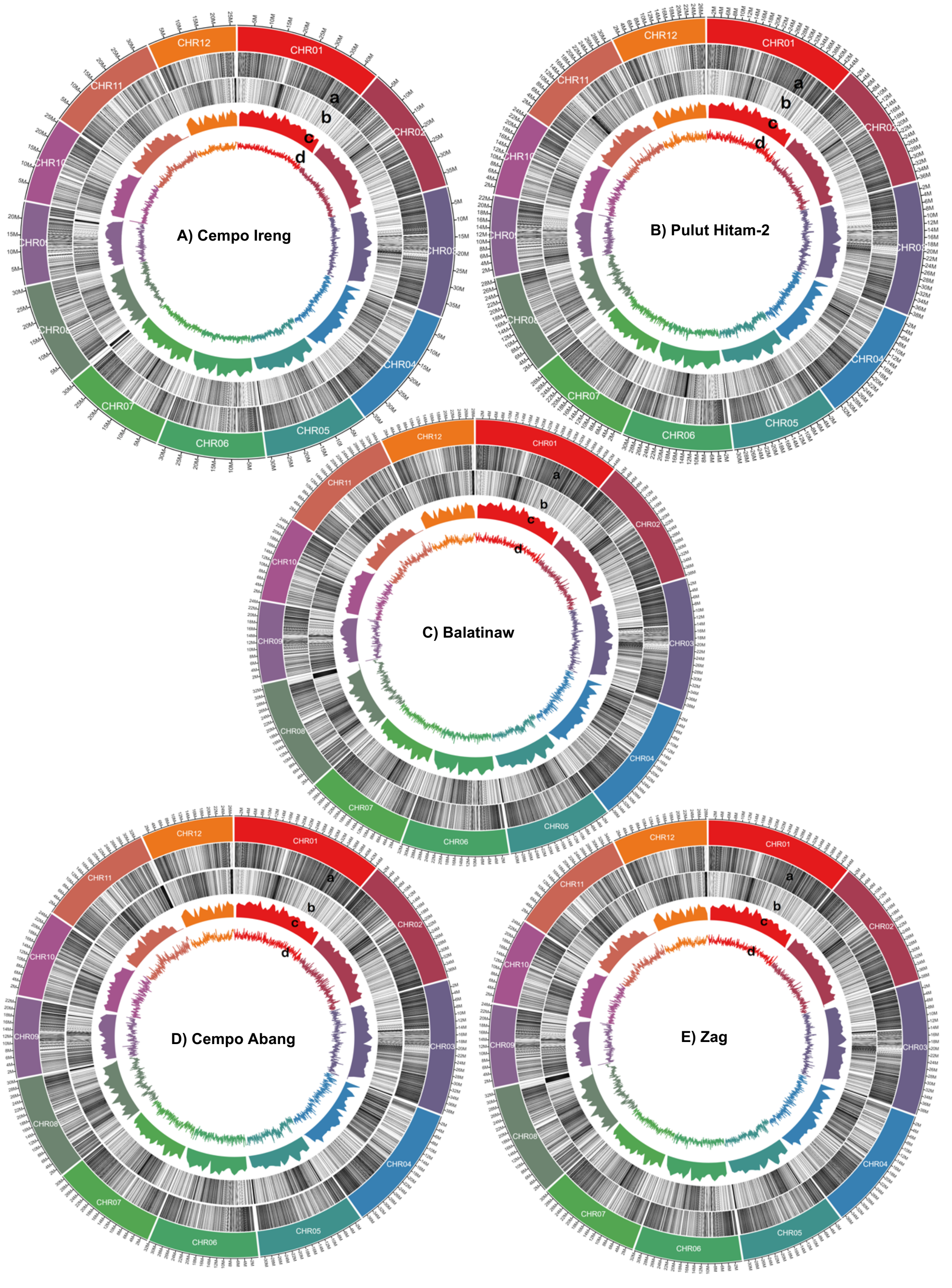

Supplementary Fig. 2. Genomic landscapes of the 12 chromosomes in the five genome assemblies of pigmented rice. Characterization of A) Cempo Ireng, B), Pulut Hitam-2, C) Balatinaw, D) Cempo Abang, and E) Zag genomes. Tracks from the outer to the inner circles represent gene density (a), transposable element (TE) density (b), SNP distribution (c), and distribution of GC content (d), respectively.

Supplementary Table 1. Summary of whole-genome sequence assembly, annotation, gene prediction, and structural variants.

	Cempo Ireng	Pulut Hitam-2	Balatinaw	Cempo Abang	Zag
**No. of CCS reads**	1,489,510	1,633,494	1,463,247	1,121,911	1,591,232
**Avg. read length (bp)**	13,700	14,273	14,446	14,421	14,516
**Total amount of data (bp)**	20,406,796,938	23,315,308,272	21,139,214,385	16,179,980,203	23,098,784,655
**Expected coverage**	~52.3×	~59.8×	~54.2×	~41.5×	~59.2×
**N50**	32,455,524	31,787,743	33,383,659	32,169,044	30,678,555
**N75**	31,037,508	28,934,096	30,817,566	30,344,714	28,207,642
**Ns / 100 kbp**	0.18	0.24	0.35	0.41	0.29
**Contigs**	19	21	26	28	23
**Genome assembly (Mbp)**	397.03	382.28	400.70	393.57	376.29
**GC content (%)**	43.7	43.6	43.7	43.7	43.5
**Repeats percentage (%)**	49.03%	47.21%	49.37%	48.74%	46.4%
**Predicted genes**	38,357	38,066	38,434	38,383	38,416
**Structural variants (Mbp)**	70.3	40.3	73.5	71.2	21.2

Supplementary Table 2. BUSCO assessment of the five genome sequence assemblies.

Accession	Completeness (%)	Complete with single-copy (%)	Complete with duplicated (%)	Fragmented (%)	Missing (%)
**Cempo Ireng**	98.5	96.3	2.2	0.2	1.3
**Pulut Hitam-2**	98.5	96.3	2.2	0.2	1.3
**Balatinaw**	98.5	96.3	2.2	0.2	1.3
**Cempo Abang**	98.4	96.3	2.1	0.2	1.4
**Zag**	98.6	96.3	2.3	0.2	1.2

Supplementary Table 3. Structural variants (SVs) distribution in pigmented rice genome sequences. The number and the total size of SVs identified in the five genomes and not shared with Nipponbare.

Chromosome	Cempo Ireng		Pulut Hitam-2		Balatinaw		Cempo Abang		Zag	
	SVs no.	SVs total size (Mbp)	SVs no.	SVs total size (Mbp)	SVs no.	SVs total size (Mbp)	SVs no.	SVs total size (Mbp)	SVs no.	SVs total size (Mbp)
1	4,198	7.9	2,134	3.7	4,447	8.4	4,351	8.4	1,029	1.7
2	3,586	6.4	1,201	2.3	3,480	6.8	3,271	6.2	761	1.3
3	2,872	4.7	1,410	2.2	3,232	5.7	3,298	5.6	651	0.6
4	2,863	5.6	1,730	4.2	2,933	5.7	3,113	6.1	1,379	3.2
5	2,489	5.3	1,558	3.5	2,440	5.2	2,264	4.8	794	2
6	3,114	7.3	1,217	2.7	3,255	7.3	3,172	7	1,258	2.6
7	3,151	5.7	1,382	2.7	3,218	5.9	2,989	5.7	747	0.8
8	2,791	6.4	1,875	5.7	2,862	6.6	2,789	6.3	1,180	3.1
9	2,230	4.5	1,071	3	2,303	4.7	2,302	4.6	648	1.3
10	2,669	5.3	1,821	3.6	2,687	5.2	2,616	5.2	118	2.7
11	3,401	6.5	1,604	3.1	3,256	6.6	3,251	6.2	1,349	1.3
12	2,903	4.8	1,792	3.6	3,063	5.4	3,226	5.1	775	0.6
**Total**	36,267	**70.3**	18,795	**40.3**	37,176	**73.5**	36,642	**71.2**	10,689	**21.2**

Supplementary Table 4. Resequencing of pigmented rice accessions. Summary of the Illumina sequencing statistics. * denotes the synthetic Illumina reads generated in silico for the PacBio sequenced genome.

Accession Name	IRGC Code	Sequencing technology	Raw Data (Gb)	Raw Read Pairs (Million)	Sequencing Coverage	Mapping Coverage (-fold)
Abor-Red-B	IRGC 10046	NovaSeq6000	7.74	47.89	35.91	27.32
Adong-Hitam	IRGC 60201	NovaSeq6000	7.06	43.52	32.64	24.36
Baisbish	IRGC 5811	NovaSeq6000	6.87	42.81	32.10	22.55
Balatinaw*	IRGC 52968	synthetic reads	4.17	40.00	30.00	22.53
Banskopi-Red	IRGC 67695	NovaSeq6000	6.64	41.46	31.10	21.15
Beni-Denia	IRGC 52627	NovaSeq6000	6.83	42.40	31.80	22.45
Beras-Merah24	NA	NovaSeq6000	6.56	40.29	30.22	21.66
Bulu-Hitam	IRGC 35570	NovaSeq6000	6.23	38.58	28.94	22.55
Cempo Abang*	NA	synthetic reads	4.17	40.00	30.00	22.72
Cempo Ireng*	NA	synthetic reads	4.17	40.00	30.00	22.68
Cempo-Merah	IRGC 35242	NovaSeq6000	7.30	44.65	33.49	22.67
Cempo-Turi	IRGC 35246	NovaSeq6000	7.05	43.54	32.66	24.38
Chakhao	IRGC 51953	NovaSeq6000	7.50	46.50	34.88	23.03
DNJ61	IRGC 8374	NovaSeq6000	6.64	41.04	30.78	21.79
DZ78	IRGC 117610	NovaSeq6000	7.21	44.84	33.63	23.90
Heenati	IRGC 8921	NovaSeq6000	7.28	45.29	33.97	24.78
Hilay	IRGC 47221	NovaSeq6000	7.39	45.89	34.42	23.19
Hwang-Mu	IRGC 1222	NovaSeq6000	7.38	45.60	34.20	23.62
Kaivari-Samba	IRGC 49732	NovaSeq6000	7.23	44.79	33.59	23.03
Kalubalawee	IRGC 15250	NovaSeq6000	5.09	31.91	23.93	16.77
Ketan-Adang	IRGC 93538	NovaSeq6000	6.84	42.67	32.00	21.59
Ketan-Hitam	IRGC 24967	NovaSeq6000	6.73	41.31	30.98	23.60
Ketan-Ireng1	IRGC 35732	NovaSeq6000	7.00	43.36	32.52	24.55
Ketan-Ireng2	IRGC 35733	NovaSeq6000	7.36	45.74	34.31	25.79
Ketan-Ireng3	IRGC 43440	NovaSeq6000	6.49	40.58	30.44	22.77
Khao-Gam-Niaw	IRGC 15016	NovaSeq6000	6.33	39.25	29.44	22.63
Khao-Khan-Nwe	IRGC 126659	NovaSeq6000	2.22	13.82	10.37	6.90
Khao-Niaw-Dam	IRGC 48143	NovaSeq6000	6.38	39.90	29.92	19.65
Khoya-Motor	IRGC 26382	NovaSeq6000	6.64	41.25	30.93	22.03
Kum-Kour	IRGC 57150	NovaSeq6000	7.50	45.89	34.42	25.79
Leukat-Hitam	IRGC 93554	NovaSeq6000	6.75	42.37	31.78	22.60
Masuran	IRGC 11997	NovaSeq6000	6.28	38.91	29.18	19.45
Ma-Zhan	IRGC 60184	NovaSeq6000	6.39	39.94	29.95	19.87
Mitak	IRGC 13592	NovaSeq6000	7.21	44.42	33.32	25.64
Nar-B-Upland	IRGC 1018	NovaSeq6000	6.52	40.67	30.50	20.73
Niaw-Dam	IRGC 48386	NovaSeq6000	6.65	80.75	60.56	23.44
Padi-Arang	IRGC 74639	NovaSeq6000	7.39	46.21	34.66	23.59
Parsan	IRGC 47309	NovaSeq6000	6.56	41.14	30.85	20.26

Periavelai-Red-Rice	IRGC 50025	NovaSeq6000	6.60	41.40	31.05	21.27
Perurutong-Nb-A	IRGC 566	NovaSeq6000	6.11	37.71	28.28	21.97
Pulut-Arang	IRGC 78905	NovaSeq6000	6.16	38.06	28.55	22.46
Pulut-Hitam1	IRGC 20083	NovaSeq6000	5.58	35.05	26.29	19.28
Pulut-Hitam2*	IRGC 110966	synthetic reads	4.17	40.00	30.00	23.45
Pulut-Hitam3	IRGC 36039	NovaSeq6000	6.37	40.20	30.15	20.21
Pulut-Hitam-Siam	IRGC 54574	NovaSeq6000	6.39	39.30	29.48	22.65
Rayada	IRGC 77208	NovaSeq6000	6.56	40.65	30.49	21.17
Rebo	IRGC 13586	NovaSeq6000	7.30	45.04	33.78	25.96
Red42	IRGC 47309	NovaSeq6000	8.16	50.81	38.11	25.83
Seneman	IRGC 53169	NovaSeq6000	6.95	43.02	32.26	24.10
Tapol	IRGC 615	NovaSeq6000	6.07	37.58	28.19	21.50
Zag*	IRGC 34388	synthetic reads	4.17	40.00	30.00	24.56

Supplementary Table 5. List of pigmented rice accessions and corresponding subpopulations. Table shows accession name, IRGC accession number, color of the grain, country of origin, and assigned subpopulation of 51 whole-genome sequenced accessions (cA1: *circum*-Aus1, cA2: *circum*-Aus2, cB: *circum*-Basmati, GJ-subtrop: *Geng-japonica* subtropical, GJ-temp: *Geng-japonica* temperate, GJ-trop1: *Geng-japonica* tropical1, GJ-trop2: *Geng-japonica* tropical2, XI-1B1: *Xian-indica* 1B1, XI-1B2: *Xian-indica* 1B2, XI-2A: *Xian-indica* 2A, XI-2B: *Xian-indica* 2B, XI-3A: *Xian-indica* 3A, XI-3B1: *Xian*-indica 3B1, XI-3B2: *Xian-indica* 3B2, XI-adm: *Xian-indica* admixed). (*) Accession sequenced with PacBio CCS from which synthetic Illumina reads were generated.

Accession Name	IRGC Label	Grain color	Country of Origin	Subpopulation
Abor-Red-B	IRGC 10046	red	India	GJ-subtrop
Adong-Hitam	IRGC 60201	black	Malaysia	GJ-trop1
Baisbish	IRGC 5811	red	Bangladesh	cA2
Balatinaw*	IRGC 52968	black	Philippines	XI-3B2
Banskopi-Red	IRGC 67695	red	India	cA2
Beni-Denia	IRGC 52627	red	India	cA2
Beras-Merah24	NA	red	Indonesia	XI-1B1
Bulu-Hitam	IRGC 35570	black	Philippines	GJ-trop1
Cempo Abang*	NA	red	Indonesia	XI-1B1
Cempo Ireng*	NA	black	Indonesia	XI-adm
Cempo-Merah	IRGC 35242	red	Indonesia	XI-3A
Cempo-Turi	IRGC 35246	red	Indonesia	GJ-trop1
Chakhao	IRGC 51953	black	India	XI-adm
DNJ61	IRGC 8374	red	Bangladesh	cA1
DZ78	IRGC 117610	red	Bangladesh	cA1
Heenati	IRGC 8921	red	Sri Lanka	cA2
Hilay	IRGC 47221	red	Philippines	XI-3B2
Hwang-Mu	IRGC 1222	red	China	XI-3B2
Kaivari-Samba	IRGC 49732	red	India	XI-adm
Kalubalawee	IRGC 15250	red	Sri Lanka	cA2
Ketan-Adang	IRGC 93538	black	Indonesia	XI-3A
Ketan-Hitam	IRGC 24967	black	Indonesia	GJ-trop1
Ketan-Ireng1	IRGC 35732	black	Indonesia	GJ-trop2
Ketan-Ireng2	IRGC 35733	black	Indonesia	GJ-trop2
Ketan-Ireng3	IRGC 43440	black	Indonesia	GJ-trop1
Khao-Gam-Niaw	IRGC 15016	black	Thailand	GJ-subtrop
Khao-Khan-Nwe	IRGC 126659	black	Thailand	XI-adm
Khao-Niaw-Dam	IRGC 48143	black	Thailand	XI-3B1
Khoya-Motor	IRGC 26382	red	Bangladesh	cB
Kum-Kour	IRGC 57150	black	Thailand	GJ-subtrop
Leukat-Hitam	IRGC 93554	black	Indonesia	GJ-trop1
Masuran	IRGC 11997	red	Sri Lanka	XI-2B
Ma-Zhan	IRGC 60184	red	China	XI-1A
Mitak	IRGC 13592	black	Indonesia	GJ-trop1
Nar-B-Upland	IRGC 1018	red	China	XI-3B2
Niaw-Dam	IRGC 48386	black	Thailand	GJ-subtrop
Padi-Arang	IRGC 74639	black	Indonesia	XI-3A

Parsan	IRGC 47309	red	Philippines	XI-adm
Periavelai-Red-Rice	IRGC 50025	red	Sri Lanka	XI-2B
Perurutong-Nb-A	IRGC 566	black	Philippines	GJ-trop1
Pulut-Arang	IRGC 78905	black	Malaysia	GJ-trop1
Pulut-Hitam1	IRGC 20083	black	Indonesia	GJ-trop2
Pulut-Hitam2*	IRGC 110966	black	Malaysia	GJ-trop1
Pulut-Hitam3	IRGC 36039	red	Malaysia	XI-3A
Pulut-Hitam-Siam	IRGC 54574	black	Philippines	GJ-trop1
Rayada	IRGC 77208	red	Bangladesh	cB
Rebo	IRGC 13586	red	Indonesia	GJ-trop1
Red42	IRGC 47309	red	Philippines	XI-1B2
Seneman	IRGC 53169	red	Philippines	GJ-trop1
Tapol	IRGC 615	black	Philippines	GJ-trop1
Zag*	IRGC 34388	red	India	GJ-temp

Supplementary Table 6. Rice accessions used for phylogenetic analysis. The table shows accession name, NCBI BioSample number, IRGC accession number, country of origin, and *O. sativa* subpopulation for 474 accessions selected from the 3K-RG as described in Wang, Mauleon, et al. (2018) and in Zhou, Chebotarov, et al. (2020) (cA1: *circum*-Aus1, cA2: *circum*-Aus2, cB: *circum*-Basmati, GJ-subtrop: *Geng-japonica* subtropical, GJ-temp: *Geng-japonica* temperate, GJ-trop1: *Geng-japonica* tropical1, GJ-trop2: *Geng-japonica* tropical2, XI-1B1: *Xian-indica* 1B1, XI-1B2: *Xian-indica* 1B2, XI-2A: *Xian-indica* 2A, XI-2B: *Xian-indica* 2B, XI-3A: *Xian-indica* 3A, XI-3B1: *Xian-indica* 3B1, XI-3B2: *Xian-indica* 3B2, XI-adm: *Xian-indica* admixed).

Accession Name	NCBI Label	IRGC Label	Country of Origin	Subpopulation
B001	ERS470219	IRGC 135900	China	GJ-temp
B004	ERS470222	IRGC 136031	Japan	GJ-temp
B006	ERS470224	IRGC 136078	Viet Nam	XI-3B2
B007	ERS470225	IRGC 136055	Viet Nam	XI-3B2
B012	ERS470230	IRGC 135815	India	XI-2B
B015	ERS470233	IRGC 135963	Romania	XI-1A
B016	ERS470234	IRGC 135829	Hungary	GJ-temp
B018	ERS470236	IRGC 135827	United States of America	GJ-trop1
B021	ERS470239	IRGC 136044	Australia	XI-3B2
B027	ERS470244	IRGC 135858	Philippines	XI-3A
B045	ERS470260	IRGC 135896	Japan	GJ-temp
B046	ERS470261	IRGC 136032	Japan	GJ-temp
B049	ERS470264	IRGC 136086	Nepal	cA1
B052	ERS470266	IRGC 135949	Madagascar	XI-2B
B073	ERS470287	NA	China	XI-3B2
B076	ERS470290	NA	China	XI-3B2
B095	ERS470307	NA	China	XI-3B2
B105	ERS470315	NA	China	XI-3B2
B113	ERS470323	NA	China	XI-3B2
B131	ERS470341	NA	China	XI-3B2
B157	ERS470367	NA	China	XI-1A
B160	ERS470370	NA	China	GJ-temp
B162	ERS470372	NA	China	GJ-temp
B164	ERS470374	NA	China	cA1
B182	ERS470387	NA	Japan	GJ-temp
B183	ERS470388	IRGC 136030	Japan	GJ-temp
B190	ERS470394	NA	Nigeria	GJ-trop1
B204	ERS470408	NA	China	GJ-temp
B208	ERS470411	NA	China	XI-1A
B210	ERS470412	NA	China	XI-1A
B212	ERS470413	NA	China	GJ-temp
B214	ERS470415	NA	China	XI-1B2
B217	ERS470418	NA	China	XI-1B2
B232	ERS470431	NA	China	XI-1B2
B241	ERS470439	NA	China	GJ-subtrop
B243	ERS470441	IRGC 135910	China	cA1
B250	ERS470448	NA	China	GJ-temp
B261	ERS470456	NA	China	XI-1A
B264	ERS470458	NA	China	XI-1A
B265	ERS470459	NA	China	XI-3B2
B266	ERS470460	NA	China	GJ-subtrop
B269	ERS470463	IRGC 136083	Japan	GJ-temp
CX10	ERS470464	IRGC 135890	China	XI-1A
CX100	ERS470465	IRGC 136020	Nepal	XI-1B2
CX101	ERS470466	IRGC 135902	China	XI-1A
CX102	ERS470467	IRGC 136053	Taiwan	XI-1A

CX104	ERS470469	IRGC 136037	Iran	cB
CX106	ERS470470	IRGC 136040	Viet Nam	GJ-trop1
CX110	ERS470475	IRGC 136062	Japan	cB
CX111	ERS470476	IRGC 135894	Egypt	GJ-trop1
CX125	ERS470491	NA	China	XI-1B2
CX129	ERS470494	IRGC 135986	Indonesia	GJ-trop2
CX133	ERS470499	NA	China	XI-1A
CX134	ERS470500	IRGC 135948	Philippines	XI-1B2
CX140	ERS470504	IRGC 136196	Japan	GJ-temp
CX141	ERS470505	IRGC 136019	Indonesia	XI-3A
CX145	ERS470509	NA	China	XI-adm
CX149	ERS470513	NA	India	cB
CX151	ERS470516	IRGC 135833	Philippines	GJ-trop1
CX160	ERS470525	IRGC 136066	India	XI-2B
CX220	ERS470547	IRGC 135941	Cote d'Ivoire	GJ-trop1
CX225	ERS470549	IRGC 135938	Philippines	XI-1B1
CX226	ERS470550	IRGC 135916	Philippines	XI-1B1
CX230	ERS470554	IRGC 135929	Philippines	XI-1B1
CX235	ERS470559	IRGC 136013	Philippines	XI-3A
CX240	ERS470564	IRGC 135859	Brazil	XI-3A
CX26	ERS470574	NA	Iran	XI-1B1
CX269	ERS470581	IRGC 135911	Brazil	GJ-trop1
CX276	ERS470587	IRGC 135939	Philippines	XI-1B1
CX305	ERS470607	IRGC 136090	China	XI-1B2
CX317	ERS470615	IRGC 135992	Malaysia	GJ-temp
CX340	ERS470624	IRGC 135907	China	XI-1B2
CX344	ERS470628	NA	China	GJ-temp
CX357	ERS470642	IRGC 136023	Philippines	XI-1B1
CX358	ERS470643	IRGC 136024	Philippines	XI-1B1
CX364	ERS470649	NA	NA	XI-1B1
CX368	ERS470653	IRGC 135996	India	cA1
CX369	ERS470654	IRGC 135947	Philippines	XI-1B2
CX381	ERS470667	IRGC 135821	Mali	XI-1B1
CX383	ERS470669	NA	China	GJ-temp
CX389	ERS470675	IRGC 135960	China	GJ-temp
CX403	ERS470689	NA	Philippines	XI-1B1
CX408	ERS804445	NA	Philippines	XI-1B2
CX409	ERS804446	NA	Philippines	XI-1B2
CX410	ERS804447	NA	Philippines	XI-1B2
CX411	ERS804448	NA	Philippines	XI-1B2
CX414	ERS804450	NA	Philippines	XI-1B2
CX416	ERS804451	NA	Philippines	XI-1B2
CX417	ERS804452	NA	Philippines	XI-1B2
CX418	ERS804453	NA	Philippines	XI-1B2
CX419	ERS804454	NA	Philippines	XI-1B2
CX420	ERS804455	NA	Philippines	XI-1B2
CX421	ERS804456	NA	Philippines	XI-1B2
CX423	ERS804458	NA	Philippines	XI-1B2
CX424	ERS804459	NA	Philippines	XI-1B2
CX54	ERS470705	NA	China	XI-3B2
CX561	ERS804465	NA	China	XI-1B1
CX578	ERS804466	NA	Philippines	GJ-temp
CX59	ERS470712	IRGC 135983	Philippines	cB
CX60	ERS470714	IRGC 135837	India	XI-1B1
CX65	ERS470718	IRGC 135884	Iran	cB
CX66	ERS470719	IRGC 136052	Iran	cB
CX72	ERS470725	IRGC 135838	Pakistan	cB

CX76	ERS470729	IRGC 135841	Sri Lanka	XI-1B1
CX83	ERS470736	IRGC 136074	Viet Nam	XI-1B1
CX90	ERS470744	IRGC 135863	Nepal	XI-1B2
CX92	ERS470746	IRGC 136063	India	XI-1B2
CX97	ERS470750	IRGC 135851	India	XI-adm
CX98	ERS470751	IRGC 135882	India	XI-2B
CX99	ERS470752	IRGC 135883	India	XI-2B
IRIS 313-10020	ERS467790	IRGC 120968	Sri Lanka	cA2
IRIS 313-10026	ERS468144	IRGC 125804	Madagascar	XI-2B
IRIS 313-10097	ERS468361	IRGC 125830	Republic of Korea	GJ-temp
IRIS 313-10109	ERS468229	IRGC 125735	Nigeria	XI-3A
IRIS 313-10114	ERS468156	IRGC 125739	Burundi	XI-2B
IRIS 313-10150	ERS467826	IRGC 126249	India	cA1
IRIS 313-10171	ERS468167	IRGC 125944	China	XI-1A
IRIS 313-10177	ERS468168	IRGC 125719	China	XI-3B2
IRIS 313-10179	ERS468170	IRGC 125755	China	XI-3B2
IRIS 313-10221	ERS468176	IRGC 125663	China	XI-3B2
IRIS 313-10234	ERS468178	IRGC 125873	Philippines	XI-1B1
IRIS 313-10237	ERS468180	NA	Philippines	XI-1B1
IRIS 313-10239	ERS468182	IRGC 125827	China	XI-3B2
IRIS 313-10392	ERS468305	IRGC 127075	Philippines	XI-1B1
IRIS 313-10477	ERS469372	IRGC 128454	China	XI-3B2
IRIS 313-10534	ERS469396	IRGC 127290	India	cA2
IRIS 313-10544	ERS469404	IRGC 127340	India	XI-2B
IRIS 313-10576	ERS469434	IRGC 127979	Sierra Leone	XI-2A
IRIS 313-10577	ERS469435	IRGC 128318	Philippines	GJ-trop1
IRIS 313-10582	ERS469440	IRGC 128440	Philippines	GJ-trop1
IRIS 313-10603	ERS469453	IRGC 127871	Bangladesh	cA2
IRIS 313-10605	ERS469455	IRGC 127348	Bangladesh	cA2
IRIS 313-10614	ERS469460	IRGC 128049	Hong Kong	XI-3B2
IRIS 313-10623	ERS469461	IRGC 127804	Nepal	cA2
IRIS 313-10642	ERS469465	IRGC 128455	Japan	GJ-temp
IRIS 313-10649	ERS469469	IRGC 127758	Philippines	GJ-trop2
IRIS 313-10657	ERS469478	IRGC 135801	Lao People's Democratic Republic	GJ-subtrop
IRIS 313-10664	ERS469484	IRGC 127163	India	XI-2A
IRIS 313-10687	ERS469504	NA	Malaysia	XI-3A
IRIS 313-10693	ERS469509	IRGC 132268	Indonesia	GJ-trop2
IRIS 313-10706	ERS469521	NA	Malaysia	XI-3A
IRIS 313-10718	ERS469524	IRGC 128073	Sri Lanka	cA2
IRIS 313-10744	ERS469552	IRGC 127811	Indonesia	GJ-trop2
IRIS 313-10748	ERS469557	IRGC 127578	Viet Nam	XI-3B2
IRIS 313-10762	ERS469572	IRGC 127207	Indonesia	XI-3A
IRIS 313-10778	ERS469586	NA	Indonesia	XI-3A
IRIS 313-10789	ERS469598	IRGC 132009	Indonesia	GJ-trop2
IRIS 313-10790	ERS469599	NA	Indonesia	GJ-trop2
IRIS 313-10793	ERS469602	IRGC 132257	Indonesia	GJ-trop1
IRIS 313-10802	ERS469622	NA	Indonesia	GJ-trop2
IRIS 313-10805	ERS469652	IRGC 127746	Indonesia	GJ-trop2
IRIS 313-10813	ERS469615	NA	Indonesia	XI-3A
IRIS 313-10814	ERS469616	IRGC 128163	Indonesia	XI-3A
IRIS 313-10816	ERS469618	IRGC 128164	Indonesia	GJ-trop2
IRIS 313-10817	ERS469619	IRGC 135796	Indonesia	GJ-trop2
IRIS 313-10824	ERS469627	IRGC 128053	Indonesia	XI-3A
IRIS 313-10834	ERS469637	IRGC 128314	India	GJ-subtrop
IRIS 313-10841	ERS469645	IRGC 128077	Indonesia	GJ-trop2
IRIS 313-10845	ERS469648	IRGC 132418	India	cA2
IRIS 313-10847	ERS469649	IRGC 127972	India	XI-2A

IRIS 313-10851	ERS469654	IRGC 132387	India	cB
IRIS 313-10852	ERS469655	IRGC 127169	India	cA1
IRIS 313-10858	ERS469661	IRGC 127131	India	XI-adm
IRIS 313-10861	ERS469664	IRGC 127134	India	cA1
IRIS 313-10863	ERS469666	IRGC 127937	India	XI-adm
IRIS 313-10870	ERS469674	IRGC 131966	India	GJ-subtrop
IRIS 313-10871	ERS469675	IRGC 127139	India	cA1
IRIS 313-10888	ERS469693	IRGC 127943	India	GJ-subtrop
IRIS 313-10889	ERS469694	NA	India	GJ-subtrop
IRIS 313-10891	ERS469697	IRGC 127148	India	cA1
IRIS 313-10892	ERS469698	IRGC 127149	India	cA1
IRIS 313-10894	ERS469700	IRGC 127150	India	cA1
IRIS 313-10895	ERS469711	IRGC 127151	India	GJ-subtrop
IRIS 313-10918	ERS469716	IRGC 135803	Philippines	GJ-trop1
IRIS 313-10922	ERS469720	IRGC 132264	Lao People's Democratic Republic	GJ-subtrop
IRIS 313-10923	ERS469722	IRGC 128256	Thailand	GJ-subtrop
IRIS 313-10927	ERS469726	IRGC 127828	Nepal	cA2
IRIS 313-10930	ERS469729	IRGC 127714	Bangladesh	cA1
IRIS 313-10936	ERS469736	IRGC 127504	Indonesia	GJ-trop2
IRIS 313-10937	ERS469737	IRGC 135788	Indonesia	XI-3A
IRIS 313-10938	ERS469738	NA	Indonesia	XI-3A
IRIS 313-10942	ERS469741	IRGC 127987	Indonesia	XI-3A
IRIS 313-10954	ERS469755	IRGC 127700	Indonesia	XI-3A
IRIS 313-10965	ERS469767	IRGC 127859	Bangladesh	cA1
IRIS 313-10975	ERS469778	IRGC 136183	Bangladesh	XI-2A
IRIS 313-10985	ERS469789	IRGC 132424	Bangladesh	XI-2A
IRIS 313-10986	ERS469790	IRGC 128048	Bangladesh	XI-2A
IRIS 313-10990	ERS469823	IRGC 127206	Philippines	XI-3B2
IRIS 313-10994	ERS469867	IRGC 135783	Philippines	GJ-trop2
IRIS 313-11005	ERS469800	IRGC 128140	Indonesia	GJ-trop2
IRIS 313-11007	ERS469803	IRGC 128141	Indonesia	GJ-trop2
IRIS 313-11016	ERS469813	IRGC 128328	Bangladesh	cA1
IRIS 313-11024	ERS469821	IRGC 127217	Pakistan	cA1
IRIS 313-11027	ERS469825	IRGC 127454	Pakistan	cA1
IRIS 313-11032	ERS469830	IRGC 127710	Pakistan	cB
IRIS 313-11037	ERS469836	IRGC 128211	Pakistan	cA1
IRIS 313-11039	ERS469838	IRGC 128093	China	XI-1A
IRIS 313-11043	ERS469842	NA	Malaysia	XI-3A
IRIS 313-11044	ERS469843	IRGC 128005	Malaysia	GJ-trop1
IRIS 313-11045	ERS469844	IRGC 128039	Malaysia	GJ-trop1
IRIS 313-11046	ERS469846	NA	Malaysia	GJ-trop1
IRIS 313-11047	ERS469847	IRGC 127977	Bangladesh	cA1
IRIS 313-11048	ERS469848	IRGC 127178	Bangladesh	cA1
IRIS 313-11050	ERS469850	IRGC 127180	Bangladesh	cA2
IRIS 313-11051	ERS469851	IRGC 127181	Bangladesh	cA2
IRIS 313-11053	ERS469853	IRGC 127183	Bangladesh	cA1
IRIS 313-11054	ERS469854	IRGC 127184	Bangladesh	cA2
IRIS 313-11055	ERS469855	IRGC 127185	Bangladesh	cA2
IRIS 313-11056	ERS469857	IRGC 127186	Bangladesh	cA2
IRIS 313-11057	ERS469858	IRGC 127187	Bangladesh	cA2
IRIS 313-11059	ERS469860	IRGC 127189	Bangladesh	cA2
IRIS 313-11062	ERS469863	IRGC 127218	Bangladesh	cB
IRIS 313-11063	ERS469864	IRGC 135741	Bangladesh	cA2
IRIS 313-11064	ERS469865	IRGC 132306	Bangladesh	cA2
IRIS 313-11083	ERS469916	IRGC 132248	Lao People's Democratic Republic	XI-3B1
IRIS 313-11089	ERS469884	IRGC 127824	Cambodia	XI-adm
IRIS 313-11094	ERS469889	IRGC 132274	Lao People's Democratic Republic	GJ-subtrop

IRIS 313-11097	ERS469892	IRGC 127478	Philippines	XI-3B2
IRIS 313-11098	ERS469893	IRGC 134993	Sierra Leone	XI-3A
IRIS 313-11102	ERS469898	NA	Liberia	GJ-trop1
IRIS 313-11103	ERS469899	IRGC 132329	Liberia	GJ-trop1
IRIS 313-11104	ERS469900	IRGC 131978	Liberia	GJ-trop1
IRIS 313-11112	ERS469909	IRGC 128308	Bangladesh	cA2
IRIS 313-11118	ERS469915	IRGC 135797	Viet Nam	XI-3B1
IRIS 313-11163	ERS469965	IRGC 127652	Bangladesh	cA2
IRIS 313-11191	ERS469980	IRGC 127763	Sri Lanka	cA2
IRIS 313-11202	ERS469993	IRGC 127920	China	GJ-temp
IRIS 313-11205	ERS469996	IRGC 128075	Bangladesh	XI-2A
IRIS 313-11218	ERS470009	IRGC 127982	Bangladesh	cB
IRIS 313-11224	ERS470016	IRGC 128130	Bangladesh	XI-2A
IRIS 313-11229	ERS470021	IRGC 128171	Bangladesh	XI-2A
IRIS 313-11238	ERS470031	NA	Brazil	GJ-trop1
IRIS 313-11239	ERS470032	IRGC 127978	Indonesia	XI-adm
IRIS 313-11241	ERS470034	IRGC 128001	Bangladesh	XI-adm
IRIS 313-11242	ERS470036	IRGC 128121	India	XI-adm
IRIS 313-11256	ERS470051	IRGC 127941	India	XI-2A
IRIS 313-11257	ERS470052	IRGC 127944	India	XI-adm
IRIS 313-11258	ERS470053	IRGC 127948	India	cB
IRIS 313-11260	ERS470055	IRGC 127950	India	XI-adm
IRIS 313-11262	ERS470058	IRGC 127952	India	XI-2A
IRIS 313-11265	ERS470061	IRGC 132242	India	cA1
IRIS 313-11267	ERS470073	IRGC 127956	India	XI-2A
IRIS 313-11273	ERS470137	IRGC 127962	India	XI-2A
IRIS 313-11277	ERS470065	IRGC 127157	India	cA2
IRIS 313-11279	ERS470067	IRGC 127967	India	XI-adm
IRIS 313-11289	ERS470078	IRGC 127971	India	cB
IRIS 313-11298	ERS470088	IRGC 127152	India	cA1
IRIS 313-11301	ERS470091	IRGC 127963	India	XI-2A
IRIS 313-11323	ERS470115	IRGC 127455	Bangladesh	cA2
IRIS 313-11338	ERS470130	IRGC 128097	Philippines	XI-3B2
IRIS 313-11345	ERS470138	IRGC 127891	Philippines	XI-3B2
IRIS 313-11350	ERS470143	IRGC 127983	India	cB
IRIS 313-11351	ERS470144	IRGC 127986	India	XI-2A
IRIS 313-11395	ERS470187	IRGC 135755	Indonesia	XI-3A
IRIS 313-11416	ERS470207	IRGC 127529	India	XI-2A
IRIS 313-11436	ERS468653	IRGC 127779	Cote d'Ivoire	GJ-trop1
IRIS 313-11453	ERS468669	IRGC 127916	India	XI-2B
IRIS 313-11461	ERS468676	IRGC 127881	India	XI-2B
IRIS 313-11467	ERS468681	IRGC 128372	Philippines	XI-3A
IRIS 313-11480	ERS468691	IRGC 127742	India	XI-1B2
IRIS 313-11483	ERS468694	IRGC 128244	Bangladesh	cA1
IRIS 313-11484	ERS468695	IRGC 127357	Bangladesh	cA2
IRIS 313-11489	ERS468700	IRGC 127482	India	cA1
IRIS 313-11512	ERS468721	IRGC 127693	Philippines	XI-3B2
IRIS 313-11513	ERS468722	IRGC 127269	Ecuador	XI-1B2
IRIS 313-11567	ERS468762	IRGC 127766	Nepal	cB
IRIS 313-11580	ERS468774	IRGC 127840	China	XI-3B2
IRIS 313-11602	ERS468789	IRGC 127597	India	cA1
IRIS 313-11617	ERS468795	IRGC 128495	India	cA1
IRIS 313-11618	ERS468796	IRGC 127879	India	cA1
IRIS 313-11625	ERS468804	IRGC 127281	Nepal	cB
IRIS 313-11626	ERS468805	IRGC 127395	Nepal	cB
IRIS 313-11630	ERS468809	IRGC 127737	Nepal	cB
IRIS 313-11635	ERS468812	IRGC 128294	Thailand	XI-3B1

IRIS 313-11642	ERS468818	IRGC 127661	India	XI-2B
IRIS 313-11643	ERS468819	IRGC 127662	India	XI-1B2
IRIS 313-11644	ERS468820	IRGC 127663	India	XI-2B
IRIS 313-11645	ERS468822	IRGC 127664	India	XI-2B
IRIS 313-11646	ERS468823	IRGC 127665	India	XI-2A
IRIS 313-11647	ERS468824	IRGC 127666	India	XI-2B
IRIS 313-11648	ERS468825	IRGC 127667	India	XI-adm
IRIS 313-11651	ERS468827	IRGC 128291	China	GJ-temp
IRIS 313-11652	ERS468828	IRGC 128303	China	GJ-temp
IRIS 313-11655	ERS468832	IRGC 127815	China	GJ-temp
IRIS 313-11656	ERS468833	IRGC 127196	Indonesia	XI-adm
IRIS 313-11657	ERS468834	NA	Nigeria	XI-2A
IRIS 313-11659	ERS468836	IRGC 132047	Sierra Leone	GJ-trop1
IRIS 313-11661	ERS468838	IRGC 131960	Bhutan	GJ-temp
IRIS 313-11663	ERS468839	IRGC 128383	Zimbabwe	XI-2B
IRIS 313-11664	ERS468840	IRGC 127288	China	XI-adm
IRIS 313-11665	ERS468842	IRGC 127464	China	XI-1A
IRIS 313-11667	ERS468844	IRGC 127580	China	XI-1A
IRIS 313-11668	ERS468845	IRGC 127771	China	XI-1A
IRIS 313-11669	ERS468846	IRGC 131986	China	XI-adm
IRIS 313-11671	ERS468847	IRGC 127313	Nepal	XI-adm
IRIS 313-11673	ERS468849	IRGC 128248	Philippines	GJ-trop1
IRIS 313-11674	ERS468850	IRGC 131971	Thailand	XI-3B1
IRIS 313-11677	ERS468852	IRGC 128286	Thailand	XI-3B1
IRIS 313-11681	ERS468856	IRGC 132426	Thailand	XI-adm
IRIS 313-11683	ERS468858	IRGC 127677	Thailand	XI-3B1
IRIS 313-11684	ERS468859	IRGC 132021	Thailand	XI-3B1
IRIS 313-11685	ERS468860	IRGC 127735	Thailand	XI-3B1
IRIS 313-11686	ERS468861	IRGC 132366	Thailand	XI-3B1
IRIS 313-11687	ERS468862	IRGC 132297	Thailand	XI-3B1
IRIS 313-11689	ERS468864	IRGC 128275	Republic of Korea	GJ-temp
IRIS 313-11691	ERS468866	IRGC 128467	Bhutan	GJ-subtrop
IRIS 313-11692	ERS468867	IRGC 127273	Taiwan	XI-1A
IRIS 313-11693	ERS468868	IRGC 127278	Taiwan	XI-3B2
IRIS 313-11700	ERS468881	IRGC 127306	Thailand	XI-3B1
IRIS 313-11704	ERS468921	NA	Thailand	XI-adm
IRIS 313-11705	ERS468932	IRGC 127303	Thailand	XI-3B1
IRIS 313-11706	ERS468935	IRGC 132001	Thailand	XI-3A
IRIS 313-11707	ERS468944	IRGC 132403	Thailand	XI-3B1
IRIS 313-11708	ERS468872	IRGC 127518	Thailand	XI-3B1
IRIS 313-11709	ERS468873	IRGC 132010	Thailand	XI-3B1
IRIS 313-11710	ERS468874	IRGC 134992	Thailand	XI-3B1
IRIS 313-11711	ERS468875	IRGC 132295	Thailand	XI-3B1
IRIS 313-11717	ERS468880	IRGC 127450	Indonesia	XI-adm
IRIS 313-11719	ERS468882	IRGC 127304	Thailand	XI-3B1
IRIS 313-11720	ERS468883	IRGC 127302	Thailand	XI-3B1
IRIS 313-11721	ERS468884	IRGC 127372	Thailand	XI-3B1
IRIS 313-11722	ERS468885	IRGC 128327	Bangladesh	XI-adm
IRIS 313-11723	ERS468886	IRGC 127320	Guinea	XI-2A
IRIS 313-11725	ERS468888	IRGC 128514	Japan	GJ-temp
IRIS 313-11727	ERS468890	IRGC 127416	China	XI-1A
IRIS 313-11728	ERS468891	IRGC 127581	China	XI-adm
IRIS 313-11730	ERS468893	IRGC 127753	China	XI-adm
IRIS 313-11731	ERS468894	IRGC 127754	China	XI-adm
IRIS 313-11732	ERS468895	IRGC 127755	China	XI-1A
IRIS 313-11733	ERS468896	IRGC 127801	China	XI-1A
IRIS 313-11734	ERS468897	IRGC 127803	China	XI-1A

IRIS 313-11736	ERS468899	IRGC 127595	Philippines	GJ-trop1
IRIS 313-11737	ERS468900	IRGC 127283	India	cA1
IRIS 313-11738	ERS468902	IRGC 127720	India	XI-2B
IRIS 313-11739	ERS468903	IRGC 128398	Ghana	GJ-trop1
IRIS 313-11740	ERS468904	IRGC 127872	Ghana	XI-3B2
IRIS 313-11741	ERS468905	IRGC 127407	Sri Lanka	XI-2B
IRIS 313-11742	ERS468906	IRGC 127480	India	cA2
IRIS 313-11744	ERS468908	IRGC 127114	China	XI-3B2
IRIS 313-11745	ERS468909	IRGC 127123	China	XI-1A
IRIS 313-11746	ERS468910	IRGC 127349	China	XI-1A
IRIS 313-11748	ERS468912	IRGC 127378	China	XI-3B2
IRIS 313-11752	ERS468915	IRGC 127584	China	XI-1A
IRIS 313-11753	ERS468916	IRGC 127894	China	XI-1A
IRIS 313-11755	ERS468918	IRGC 131961	Liberia	GJ-trop1
IRIS 313-11756	ERS468919	IRGC 131970	Madagascar	GJ-trop1
IRIS 313-11758	ERS468922	IRGC 131987	Cote d'Ivoire	XI-2A
IRIS 313-11759	ERS468923	IRGC 131995	Cote d'Ivoire	GJ-trop1
IRIS 313-11761	ERS468925	IRGC 127683	Cote d'Ivoire	XI-2B
IRIS 313-11762	ERS468926	IRGC 127682	Madagascar	XI-2A
IRIS 313-11764	ERS468928	IRGC 127697	Liberia	XI-adm
IRIS 313-11765	ERS468929	IRGC 127696	Liberia	cB
IRIS 313-11772	ERS468933	IRGC 128452	Madagascar	XI-2B
IRIS 313-11786	ERS468942	IRGC 128411	Gambia	XI-3B1
IRIS 313-11787	ERS468943	IRGC 127845	Gambia	XI-2A
IRIS 313-11788	ERS468945	IRGC 127247	Philippines	GJ-temp
IRIS 313-11789	ERS468946	IRGC 127774	Madagascar	XI-2B
IRIS 313-11790	ERS468947	IRGC 127883	Madagascar	GJ-trop2
IRIS 313-11791	ERS468948	IRGC 127885	Madagascar	XI-2B
IRIS 313-11794	ERS468971	IRGC 127867	Madagascar	XI-2B
IRIS 313-11795	ERS468982	IRGC 127291	China	XI-1A
IRIS 313-11796	ERS468993	IRGC 127345	China	XI-1A
IRIS 313-11797	ERS469003	IRGC 128289	China	XI-1A
IRIS 313-11799	ERS469024	IRGC 127366	China	XI-1A
IRIS 313-11800	ERS469031	IRGC 136098	China	GJ-temp
IRIS 313-11801	ERS468950	IRGC 127368	China	XI-adm
IRIS 313-11802	ERS468951	IRGC 127460	China	XI-1A
IRIS 313-11804	ERS468953	IRGC 128441	China	XI-1A
IRIS 313-11805	ERS468954	IRGC 127895	China	XI-1A
IRIS 313-11807	ERS468956	IRGC 127757	Colombia	XI-adm
IRIS 313-11809	ERS468958	IRGC 128387	Kenya	cA1
IRIS 313-11810	ERS468959	IRGC 132036	Kenya	XI-3B1
IRIS 313-11811	ERS468961	IRGC 127632	Kenya	XI-adm
IRIS 313-11812	ERS468962	IRGC 127175	Kenya	XI-3B1
IRIS 313-11813	ERS468963	IRGC 132372	Kenya	XI-3B1
IRIS 313-11814	ERS468964	IRGC 127523	Kenya	XI-3B1
IRIS 313-11815	ERS468965	IRGC 127633	Kenya	XI-3B1
IRIS 313-11817	ERS468967	IRGC 128085	Myanmar	XI-adm
IRIS 313-11819	ERS468969	IRGC 132362	Myanmar	XI-3B1
IRIS 313-11820	ERS468970	IRGC 127709	Myanmar	XI-3B1
IRIS 313-11821	ERS468972	IRGC 127096	India	XI-2B
IRIS 313-11822	ERS468973	IRGC 135738	India	XI-2B
IRIS 313-11823	ERS468974	IRGC 127223	India	XI-adm
IRIS 313-11824	ERS468975	IRGC 127317	India	XI-2B
IRIS 313-11825	ERS468976	IRGC 135753	India	cB
IRIS 313-11832	ERS468984	IRGC 131964	Thailand	GJ-subtrop
IRIS 313-11842	ERS468995	NA	Thailand	XI-3A
IRIS 313-11887	ERS469057	IRGC 127434	Philippines	XI-1B1

IRIS 313-11897	ERS469039	IRGC 128431	Thailand	GJ-subtrop
IRIS 313-11900	ERS469042	IRGC 128435	Thailand	GJ-subtrop
IRIS 313-11920	ERS469064	IRGC 127386	Thailand	XI-2A
IRIS 313-11924	ERS469069	IRGC 128400	Thailand	GJ-subtrop
IRIS 313-11929	ERS469074	IRGC 127385	Philippines	GJ-trop1
IRIS 313-11941	ERS469086	IRGC 128394	Burkina Faso	XI-1B2
IRIS 313-11945	ERS469090	IRGC 127598	Bangladesh	XI-2A
IRIS 313-11963	ERS469108	IRGC 127491	India	cA2
IRIS 313-11978	ERS469124	IRGC 127440	Philippines	XI-1B1
IRIS 313-12029	ERS469163	IRGC 132278	Lao People's Democratic Republic	GJ-subtrop
IRIS 313-12048	ERS469180	IRGC 127564	Indonesia	XI-3A
IRIS 313-12057	ERS469189	IRGC 127116	China	XI-3B2
IRIS 313-12058	ERS469190	IRGC 132406	Cambodia	XI-3B1
IRIS 313-12071	ERS469203	IRGC 128406	Lao People's Democratic Republic	GJ-subtrop
IRIS 313-12094	ERS469210	IRGC 127496	Bangladesh	cB
IRIS 313-12118	ERS469218	IRGC 127691	Madagascar	XI-2B
IRIS 313-12129	ERS469223	IRGC 128391	Lao People's Democratic Republic	GJ-subtrop
IRIS 313-12134	ERS469228	IRGC 135802	Lao People's Democratic Republic	GJ-subtrop
IRIS 313-12135	ERS469229	IRGC 127726	Malaysia	XI-1B1
IRIS 313-12141	ERS469232	IRGC 132399	Bangladesh	cA2
IRIS 313-12183	ERS469248	IRGC 127796	Nepal	cA1
IRIS 313-12228	ERS469267	IRGC 128474	Lao People's Democratic Republic	GJ-subtrop
IRIS 313-12234	ERS469271	IRGC 127124	China	XI-1B2
IRIS 313-12258	ERS469285	IRGC 128283	Lao People's Democratic Republic	GJ-subtrop
IRIS 313-12265	ERS469335	IRGC 128352	Lao People's Democratic Republic	GJ-subtrop
IRIS 313-12275	ERS469293	IRGC 127590	China	XI-3B2
IRIS 313-12289	ERS469307	IRGC 132304	Myanmar	GJ-subtrop
IRIS 313-12307	ERS469320	IRGC 132034	Lao People's Democratic Republic	GJ-subtrop
IRIS 313-12312	ERS469325	IRGC 128482	Lao People's Democratic Republic	GJ-subtrop
IRIS 313-12321	ERS469328	IRGC 127557	Lao People's Democratic Republic	GJ-subtrop
IRIS 313-12323	ERS469330	IRGC 135563	Lao People's Democratic Republic	GJ-subtrop
IRIS 313-12349	ERS469349	IRGC 127592	Lao People's Democratic Republic	GJ-subtrop
IRIS 313-12351	ERS469351	IRGC 135759	Lao People's Democratic Republic	GJ-subtrop
IRIS 313-15908	ERS467765	IRGC 126955	Colombia	cA2
IRIS 313-15910	ERS467767	IRGC 126956	United States of America	GJ-trop1
IRIS 313-7685	ERS468582	IRGC 122089	Philippines	XI-1B1
IRIS 313-7688	ERS468560	IRGC 122091	Philippines	XI-1B1
IRIS 313-7689	ERS468576	IRGC 122092	Philippines	XI-1B1
IRIS 313-7807	ERS468569	IRGC 122284	Senegal	XI-1B1
IRIS 313-7816	ERS468567	IRGC 122288	Senegal	XI-1B1
IRIS 313-7826	ERS468566	IRGC 122292	Senegal	XI-1B1
IRIS 313-7902	ERS468619	IRGC 122101	Philippines	GJ-trop1
IRIS 313-7914	ERS468607	IRGC 122115	Cote d'Ivoire	GJ-trop1
IRIS 313-8173	ERS468510	IRGC 121959	United States of America	GJ-trop1
IRIS 313-8312	ERS468239	IRGC 131932	Malaysia	XI-3A
IRIS 313-8321	ERS467775	IRGC 120894	Bangladesh	cA2
IRIS 313-8326	ERS467814	IRGC 117494	India	cB
IRIS 313-8341	ERS467930	IRGC 121599	Viet Nam	XI-3B2
IRIS 313-8391	ERS468241	NA	Burkina Faso	XI-2A
IRIS 313-8407	ERS468242	NA	Malaysia	XI-3A
IRIS 313-8436	ERS468435	IRGC 126307	Indonesia	GJ-trop2
IRIS 313-8493	ERS468246	IRGC 128537	Indonesia	XI-3A
IRIS 313-8530	ERS468211	IRGC 126199	India	XI-2A
IRIS 313-8557	ERS468247	IRGC 131956	Malaysia	XI-3A
IRIS 313-8568	ERS467875	IRGC 125619	India	XI-2B
IRIS 313-8595	ERS468250	IRGC 126240	Madagascar	XI-2B
IRIS 313-8606	ERS468213	IRGC 131926	India	XI-3A

IRIS 313-8616	ERS467956	IRGC 131939	Philippines	XI-1B1
IRIS 313-8641	ERS467862	IRGC 120876	Bangladesh	cA1
IRIS 313-8681	ERS468253	NA	Guinea	XI-3A
IRIS 313-8703	ERS468256	IRGC 124432	Bangladesh	XI-2A
IRIS 313-8721	ERS467780	IRGC 120969	Bangladesh	cA2
IRIS 313-8725	ERS468259	IRGC 126262	Indonesia	XI-3A
IRIS 313-8755	ERS468320	IRGC 126120	Japan	GJ-temp
IRIS 313-8813	ERS467771	IRGC 117425	India	cB
IRIS 313-8864	ERS467823	IRGC 121069	Bangladesh	cA1
IRIS 313-8925	ERS467992	IRGC 125813	Sri Lanka	XI-2B
IRIS 313-8948	ERS467998	IRGC 126974	Philippines	XI-3B1
IRIS 313-8988	ERS468007	IRGC 125672	India	XI-2B
IRIS 313-9020	ERS468013	IRGC 126983	Thailand	XI-3B1
IRIS 313-9039	ERS467824	IRGC 121441	Sri Lanka	XI-2B
IRIS 313-9044	ERS467876	IRGC 125971	Philippines	XI-1B1
IRIS 313-9048	ERS467897	IRGC 125930	Bhutan	GJ-subtrop
IRIS 313-9066	ERS468017	IRGC 125659	Bangladesh	XI-adm
IRIS 313-9067	ERS468018	IRGC 125681	Bangladesh	XI-2A
IRIS 313-9121	ERS468027	IRGC 125802	Thailand	XI-2A
IRIS 313-9156	ERS468219	IRGC 126200	Bangladesh	XI-2A
IRIS 313-9262	ERS468048	IRGC 125785	Bangladesh	XI-2A
IRIS 313-9294	ERS468283	IRGC 131929	Gambia	XI-adm
IRIS 313-9301	ERS467891	IRGC 126068	Indonesia	GJ-trop2
IRIS 313-9409	ERS468225	IRGC 131937	Malaysia	XI-3A
IRIS 313-9422	ERS467785	IRGC 120915	Bangladesh	cA2
IRIS 313-9626	ERS467787	IRGC 121005	Bangladesh	cA2
IRIS 313-9682	ERS467908	IRGC 135548	Bangladesh	cB
IRIS 313-9922	ERS468126	IRGC 125778	Republic of Korea	XI-1B2
IRIS 313-9966	ERS468133	IRGC 125713	Colombia	XI-1B2

Supplementary S2

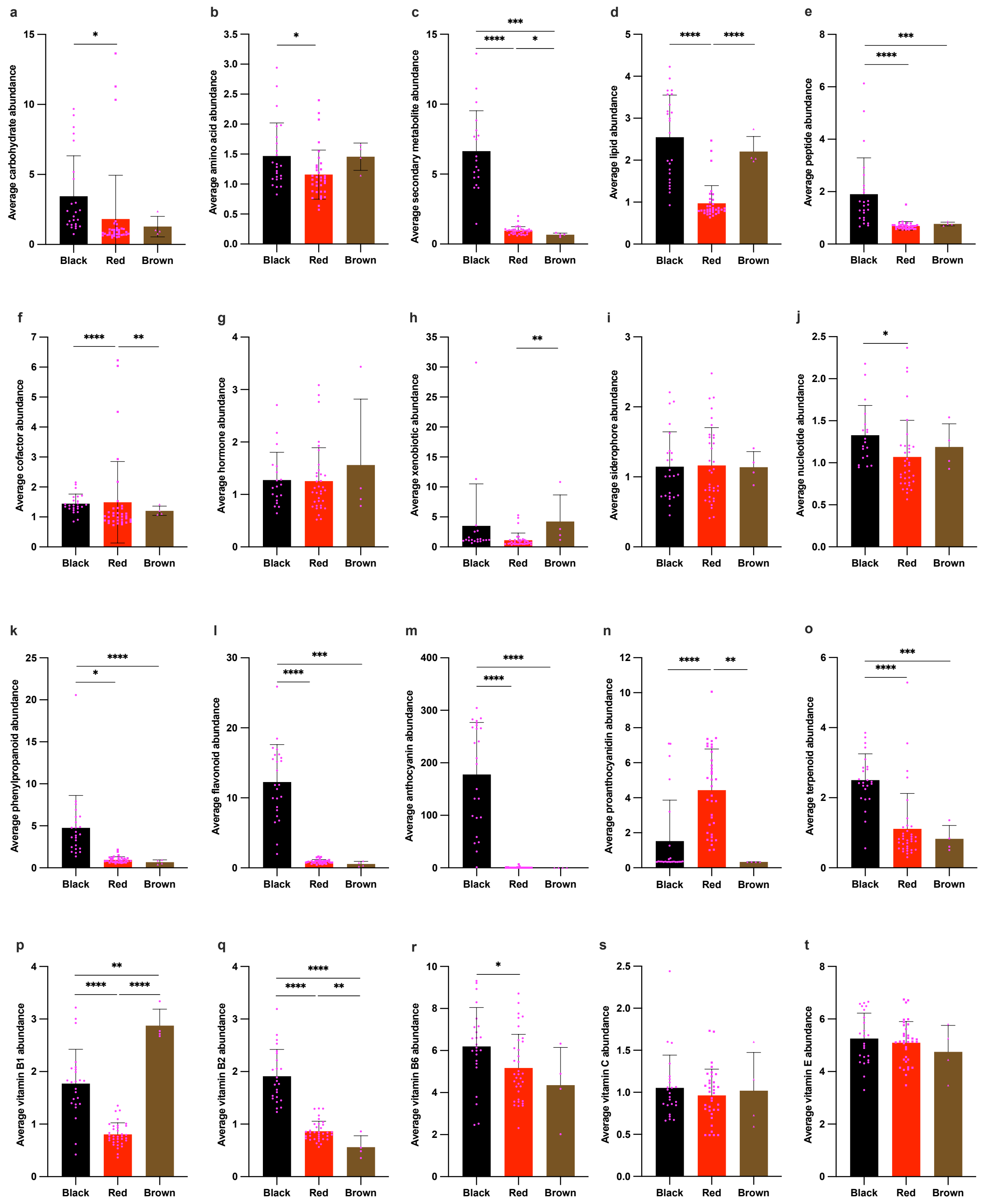

Supplementary Fig. 3. Comparison of the average metabolite abundance among pigmented rice varieties. Unpaired *t*-test comparing black (*n*=24) and red (*n*=35), black and brown (*n*=4), and red and brown rice. a) Carbohydrates. b) Amino acids. c) Secondary metabolites. d) Lipids. e) Peptides. f) Cofactors. g) Hormones. h) Xenobiotics. i) Siderophores. j) Nucleotides. k) Phenylpropanoids. l) Flavonoids. m) Anthocyanins. n) Proanthocyanidins. o) Terpenoids. p) Vitamin B1, q) Vitamin B2, r) Vitamin B6, s) Vitamin C, and t) Vitamin E. Data are presented as mean values with the error bars denoting 95% confidence intervals. Asterisks on the graph represent significant differences: *, p <0.05; **, p <0.01; ***, p <0.001; ****, p <0.0001.

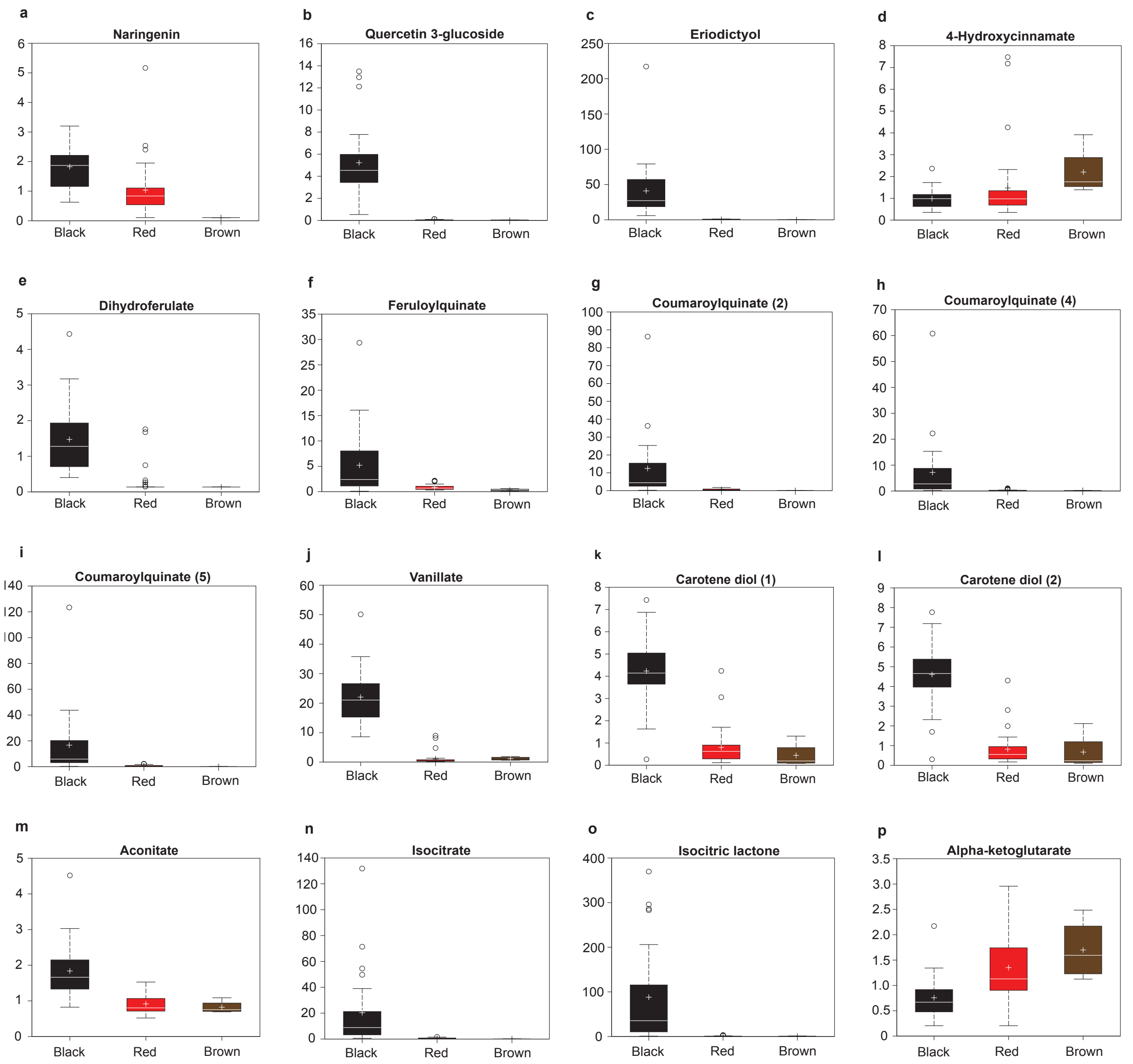

Supplementary Fig. 4. Box plots of selected secondary metabolites differing significantly by the grain color variable. Black (*n*=24), red (*n*=35), and brown (*n*=4) rice varieties. a) Naringenin. b) Quercetin 3-glucoside. c) Eriodictyol. d) 4-hydroxycinnamate. e) Dihydroferulate. f) Feruloylquinate (2). g) Coumaroylquinate (2). h) Coumaroylquinate (4). i) Coumaroylquinate (5). j) Vanillate. k) Carotene diol (1). l) Carotene diol (2). m) Aconitate. n) Isocitrate. o) Isocitrate lactone. p) Alpha-ketoglutarate. Box denotes 25th and 75th percentiles; line within box denotes 50th percentile; the solid bar across the box represents the median value while the "+" symbol indicates the mean value; whisker denotes standard deviation and outliers are represented by circles above the box.

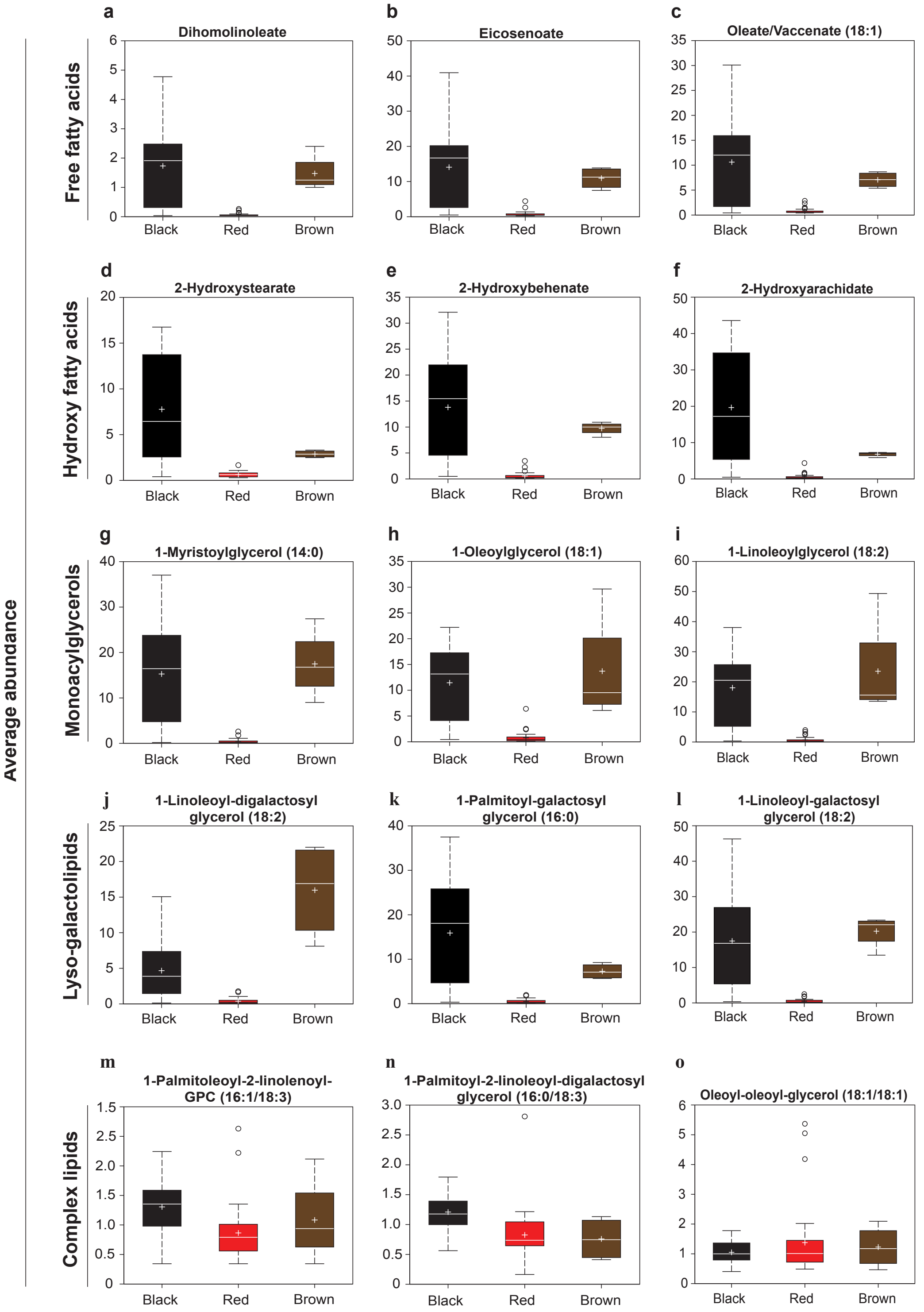

Supplementary Fig. 5. Box plots showing examples of lipid species differentially expressed in the different types of pigmented rice. Black (*n*=24), red (*n*=35), and brown (*n*=4) rice varieties. Five lipid classes, including free fatty acids (a–c), hydroxy fatty acids (d–f), monoacylglycerols (g–i), *lyso*-galactolipids (j–l), and complex lipids (m–o). Box denotes 25th and 75th percentiles; line within box denotes 50th percentile; the solid bar across the box represents the median value while the "+" symbol indicates the mean value; whisker denotes standard deviation and outliers are represented by circles above the box.

Supplementary S3

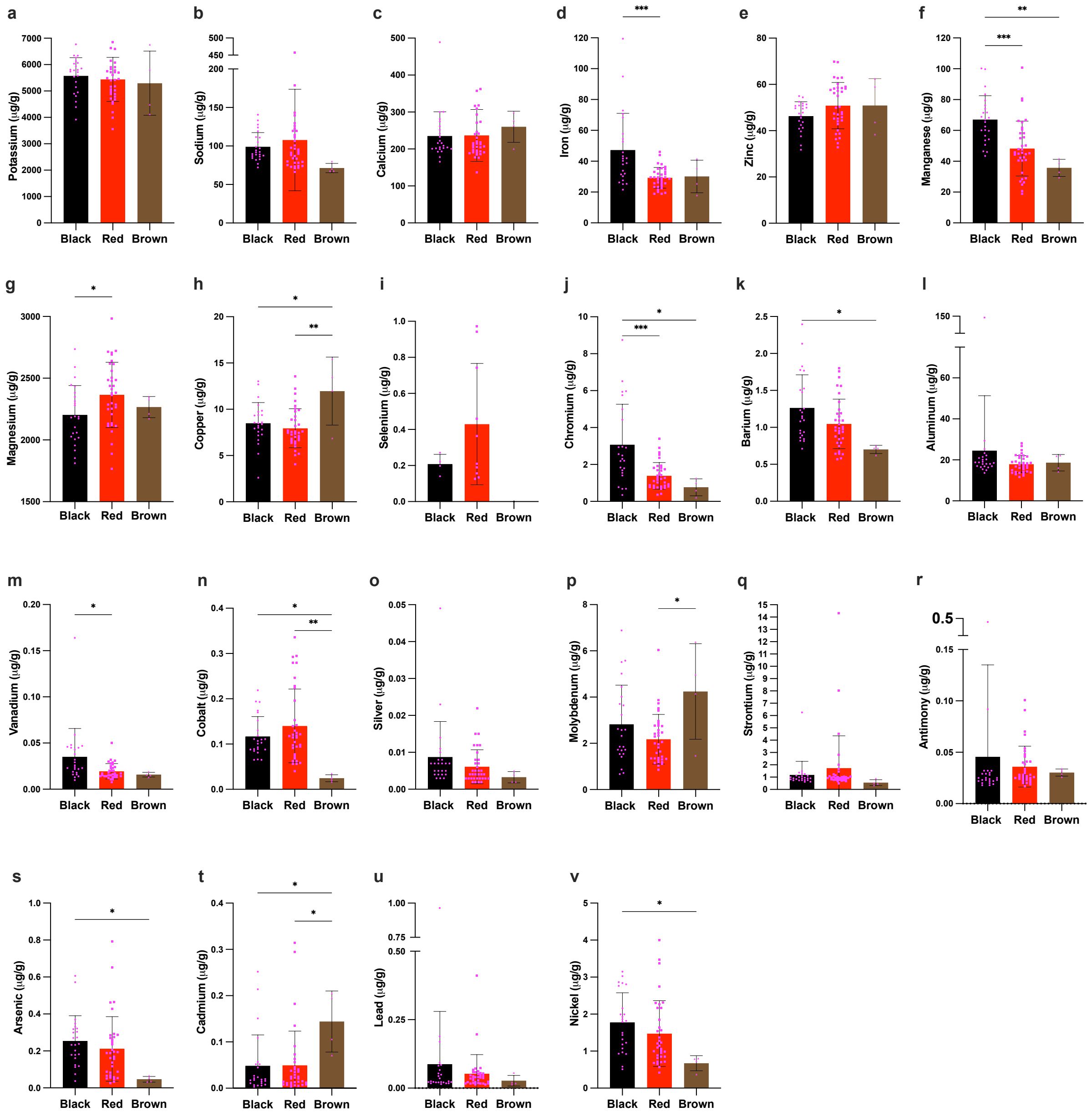

Supplementary Fig. 6. Metal ion profiling of the pigmented rice. One-way ANOVA test was performed to compare the metal ion contents of the black (*n*=24), red (*n*=35), and brown (*n*=4) rice samples. a) Potassium. b) Sodium. c) Calcium. d) Iron. e) Zinc. f) Manganese. g) Magnesium. h) Copper. i) Selenium. j) Chromium. k) Barium. l) Aluminum. m) Vanadium. n) Cobalt. o) Silver. p) Molybdenium. q) Strontium. r) Antimony. s) Arsenic. t) Cadmium. u) Lead. v) Nickel. Data are presented as mean values with the error bars denoting 95% confidence intervals. Asterisks indicate significant differences: *, p <0.05; **, p <0.01; and ***, p <0.001.

Relationship between grain pigments and metal ions

To investigate relationship between pigment enrichment in the rice grain and metal ion concentration, we performed a correlation test between anthocyanin or proanthocyanidins and the 22 trace metal ion concentrations using GraphPad Prism 9.3.1. Pearson's correlation coefficients (R) and P-values (P) were calculated. The correlation was considered to be significant at 0.05 level. The abundance of anthocyanin was positively correlated with Fe, Mn, V, Cr, Mo, and B and negatively correlated with Mg. However, the abundance of proanthocyanidins was negatively correlated with Fe, Cr, and, Mo concentrations (Supplementary File 5). These inverse relationships detected for Fe, Cr, and Mo and anthocyanin/ proanthocyanidins were not previously reported for whole grain rice varieties and merits further mechanistic investigation.

Supplementary S4

Methods

Embryogenic callus induction and regeneration. Mature dry seeds of Cempo Ireng (local to the Yogyakarta region, Indonesia) were used in this study. The seeds were dehusked and sterilized with 70% ethanol for 1 min and 30% (v/v) commercial bleach (5.25% sodium hypochlorite) for 40 min with continuous shaking, and then rinsed five times with sterilized distilled water and dried on Whatman paper for 5 min. For callus induction, 36 seeds were inoculated per Petri dish on the callus-induction media 2N6 or 2NBK (modified from the protocol developed by Hiei and Komari [11] and incubated at 30°C and 32°C in the dark or under continuous light for 7 d. All culture media components and preparations are detailed in Supplementary S6. The use of 2NBK medium at 32°C under continuous light were selected as the best conditions for callus induction based on the induced callus mass and quality.

The scutella were subcultured onto fresh 2NBK medium for another seven days under the same conditions. The embryogenic calli were induced by subculturing the calli on nNBKC for five days at 32°C. Ten different regeneration media were developed and tested for shoot induction based on MS medium and different growth regulator combinations (Supplementary S6). The embryogenic calli were selected and placed on the tested media at 32°C under continuous light for 14 days. The regeneration frequency was calculated based on the number of regenerated shoots compared with the total number of calli. For the development of roots, the regenerated shoots were transferred into magenta boxes containing MSRO rooting medium under the same temperature and light conditions.

Agrobacterium-mediated transformation. The binary vector (pRGEB32) used in this study contains *Hygromycin phosphotransferase* (*Hpt*) as a selectable marker under the control of the *CaMV 35S* promoter, and the *Cas9* gene under the control of the rice *Ubiquitin* promoter. The vector was transferred into *Agrobacterium tumefaciens* strain EHA105 by electroporation in a Bio-Rad Laboratories *Escherichia coli* pulser. Bacterial culture and transformation were conducted according to Hiei and Komari [11], with some modifications. After the co-cultivation step, the calli were placed on the first selection medium (NBKCH20) for 14 days and were then moved to the second selection medium (nNBKCH40) for five days. The calli were incubated in all steps from callus induction to the rooting stage at 32°C and under continuous light, except for the co-cultivation step, which took place at 25°C in the dark. The embryogenic calli were placed on the R8H5 medium containing 5 mg hygromycin for selection. The regenerated shoots were grown on the rooting medium (MSROH5), after which the seedlings were acclimatized in the soil and greenhouse conditions.

Genomic DNA extraction and genotyping. Total genomic DNA was extracted from approximately 0.5 g of fresh leaves from the transformed plants using the DNAquick Plant System (Tiangen Biotech), according to the manufacturer's protocol. A PCR screening was performed to amplify 240 bp of the CRISPR transgene using Phusion High-Fidelity DNA Polymerase (Thermo Fisher Scientific) and the Cas9-F7/Nos-R7 primer pair (Supplementary Table 8). The PCR products were analyzed by gel electrophoresis on 1% agarose gels.

Phenotyping of CRISPR/Cas9-targeted Cempo Ireng mutants. The genome-engineered plants and the wild-type controls were grown in the KAUST greenhouse rooms at 28°C under natural sunlight. Fifteen random homozygous

mutants were selected (5 plants per target) and 5 wild-type controls for detailed agronomic trait analysis. The heading date was recorded as the first day on which the first panicle emerged. The plant height was measured on the same day using the measuring scale. The plants were grown until fully mature, at which point the plant tillers were counted, the panicles were harvested, the number of filled and unfilled spikelet were counted in each panicle and based on that seed setting rate (the ratio of number of filled grains to total number of spikelets) was calculated. The rough, paddy rice grain length and width were measured by ImageJ software, and 1000 paddy grains had husk removed and were cleaned prior to weight measurements with a sensitive electronic balance (0.001 g sensitivity). The total yield per plant was measured as the total grams of grain collected per plant. All data were statistically analyzed by unpaired *t*-test using GraphPad Prism 9.3.1. and significant difference were indicated by asterisks: *, $p<0.05$; **, $p<0.01$; ***, $p<0.001$; ****, $p<0.0001$.

Supplementary S5

Results

Establishment of a regeneration and transformation system for BR

To establish a regeneration protocol for Cempo Ireng, we tested callus inducibility using the established protocol for *japonica* and *indica* rice developed by Hiei and Komari [11] with some modification. We used the mature grains of Cempo Ireng for *in vitro* callus induction. We tested the medium we developed (CIM57) and another two media developed by Hiei and Komari [11] with our modifications (see Supplementary S6, below), called 2N6 and 2NBK. We also tested two different incubation temperatures (30°C and 32°C) and two different light conditions (complete darkness and continuous light).

No callus was induced using CIM57 supplemented with dicamba and 1-naphthaleneacetic acid (NAA) growth regulators; however, a typical yellowish and friable callus mass emerged from the grain using media containing 2,4-dichlorophenoxyacetic acid (2N6 and 2NBK) under all test conditions (Supplementary Fig. 7e). We observed an increase in the number of induced calli when the temperature was increased to 32°C and in the presence of light. Callus induction frequency varied between 26.3% and 30.6% across the tested conditions, with the highest callus induction frequency obtained using 2NBK at 32°C in light (Supplementary Fig. 7e). Based on these results, we selected 2NBK as the best callus-induction medium and 32°C and continuous light as the conditions for the further regeneration and rooting stages (Supplementary Fig. 7).

Somatic embryos were successfully induced on the nNBKC medium containing a high concentration of sorbitol (55 g/L) and sucrose (20 g/L) (Supplementary S6). We tested ten different Murashige and Skoog (MS) basal media supplemented with different concentrations of various growth regulators (R1–R10) for their shoot induction abilities (Supplementary Fig. 7f, Supplementary S6). We did not achieve regeneration using R1, R5, and R7; however, the other seven media induced green shoots after 12–16 days of cultivation, with frequencies ranging from 33.3% to 83.3%. The highest regeneration frequency (83.3%) was achieved using R8 medium, which contained a 1:2 combination of NAA and BAP (Supplementary Fig. 7f). The shoots were healthy and developed a good root system on MSRO rooting medium for maturation into a seedling. For acclimatization, the seedlings were transferred into a greenhouse room at 28°C.

Next, we used our optimized regeneration protocol to establish a genetic transformation protocol. We tested the *Agrobacterium tumefaciens*–mediated protocol developed by Hiei and Komari [11] to transform proliferating Cempo Ireng calli. We used the pRGEB32 vector harboring hygromycin as a selectable marker and delivered it into *Agrobacterium tumefaciens* strain EHA105. We transfected the induced calli and selected them using 20 mg/L hygromycin for 14 days and 40 mg/L hygromycin for five days (Supplementary Fig. 8b-c). We observed no shoot regeneration of the transformed calli in the presence of a high concentration of hygromycin (50 mg/L). To determine the optimal hygromycin concentration for selection of transformed calli, we tested different levels of hygromycin (5–50 mg/L), and found that 5 mg/L was sufficient for selection of transformed shoots (Supplementary Fig. 8d). Using

these conditions, we found that 78.7% of the regenerated plants had successfully integrated T-DNA sequence into their genome.

Supplementary S6

Media Setup:

CIM57: Add 4.3 g Murashige and Skoog (MS) basal medium, 30 g sucrose, 1 g casein hydrolysate. Adjust pH to 5.8 and add 4 g agar and 3 g Gelzan. Autoclave at 121°C for 20 min, cool to 60°C, then add 2 mg of filter-sterilized dicamba and 10 mg of filter-sterilized $AgNO_3$.

2N6: Add 100 mL of 10× N6 major salts, 10 mL of 100× N6 minor salts, 10 mL of 100× N6 vitamins, 10 mL of 100× FeEDTA, and 20 mL of 100 mg 2,4- dichlorophenoxyacetic acid (2,4-D) to 800 mL distilled water. Dissolve 0.5 g L-proline, 0.5 g casein hydrolysate, 0.1 g myoinositol, and 30 g sucrose in the solution, then make up the volume to 900 mL. Adjust pH to 5.8 and add 4 g phytagel. Autoclave at 121°C for 20 min, cool to 60°C, then add 100 mL of 10× AA amino acids (pH 5.8).

2NBK: Add 100 mL of 10× N6K major salts, 10 mL of 100× B5 minor salts, 10 mL of 100× B5 vitamins, 10 mL of 100× FeEDTA, and 20 mL of 100 mg 2,4-D to 800 mL distilled water. Dissolve 0.5 g L-proline, 0.5 g casein hydrolysate, and 30 g maltose and make up the volume to 900 mL. Adjust pH to 5.8 and add 4 g phytagel. Autoclave at 121°C for 20 min, cool to 60°C, then add 100 mL of 10× AA amino acids (pH 5.8).

NBKCH20: Add 0.4 mL of 50 mg/mL hygromycin B, and 1 mL of 200 mg/mL timentin to the autoclaved 1L 2NBK medium after cooling to 60°C.

nNBKC: Add 100 mL of 10× N6K major salts, 10 mL of 100× B5 minor salts, 10 mL of 100× B5 vitamins, 10 mL of 100× FeEDTA, 10 mL of 100 mg 2,4-D, 0.5 mg 1-naphthaleneacetic acid (NAA), and 0.1 mg 6-benzylaminopurine (BAP) to 800 mL distilled water. Dissolve 0.5 g L-proline, 0.5 g casein hydrolysate, 20 g sucrose, 55 gm D-sorbitol, and make up the volume to 1000 mL. Adjust pH to 5.8 and add 5 g phytagel. Autoclave at 121°C for 20 min, cool to 60°C.

nNBKCH40: Add 0.8 mL of 50 mg/mL hygromycin, and 1 mL of 200 mg/mL timentin to the autoclaved 1L nNBKC medium after cooling to 60°C.

R1: Add 4.3 g MS basal medium, 30 g sucrose, 0.1 mg gibberellic acid (GA), and 0.5 mg BAP. Adjust pH to 5.8, add 4 g phytagel, and autoclave at 121°C for 20 min.

R2: Add 4.3 g MS basal medium, 30 g sucrose, 0.5 mg BAP, 0.5 mg thidiazuron, 0.5 mg kinetin, and 0.5 mg NAA. Adjust pH to 5.8, add 4 g phytagel, and autoclave at 121°C for 20 min.

R3: Add 4.3 g MS basal medium, 30 g sucrose, 2 mg BAP, and 0.5 mg kinetin. Adjust pH to 5.8, add 4 g phytagel, and autoclave at 121°C for 20 min.

R4: Add 4.3 g MS basal medium, 30 g sucrose, 2 mg BAP, and 0.5 mg thidiazuron. Adjust pH to 5.8, add 4 g phytagel, and autoclave at 121°C for 20 min.

R5: Add 4.3 g MS basal medium, 30 g sucrose, 0.5 mg thidiazuron, and 2 mg kinetin. Adjust pH to 5.8, add 4 g phytagel, and autoclave at 121°C for 20 min.

R6: Add 4.3 g MS basal medium, 30 g sucrose, 1 mg BAP, 0.5 mg kinetin, and 0.5 mg NAA. Adjust pH to 5.8, add 4 g phytagel, and autoclave at 121°C for 20 min.

R7: Add 4.3 g MS basal medium, 30 g sucrose, 1 mg BAP, 1 mg thidiazuron, and 0.5 mg NAA. Adjust pH to 5.8, add 4 g phytagel, and autoclave at 121°C for 20 min.

R8: Add 4.3 g MS basal medium, 30 g sucrose, 2 mg BAP, and 1 mg NAA. Adjust pH to 5.8, add 4 g phytagel, and autoclave at 121°C for 20 min.

R9: Add 4.3 g MS basal medium, 30 g sucrose, 1 mg kinetin, and 1 mg NAA. Adjust pH to 5.8, add 4 g phytagel, and autoclave at 121°C for 20 min.

R10: Add 4.3 g MS basal medium, 30 g sucrose, 1 mg BAP, 1 mg kinetin, and 1 mg NAA. Adjust pH to 5.8, add 4 g phytagel, and autoclave at 121°C for 20 min.

R8H5: Add 0.1 mL of 50 mg/mL hygromycin B to the autoclaved 1L R8 medium after cooling to 60°C.

MSRO: Add 2.2 g MS basal medium, 10 g sucrose, and 0.5 g casein hydrolysate to 950 mL distilled water. Adjust pH to 5.8 and add 4 g phytagel. Autoclave at 121°C for 20 min, cool to 60°C.

MSROH5: Add 0.1 mL of 50 mg/mL hygromycin B to the autoclaved 1L MSRO medium after cooling to 60°C.

Preparation of stock solutions: see Hiei and Komari [11]

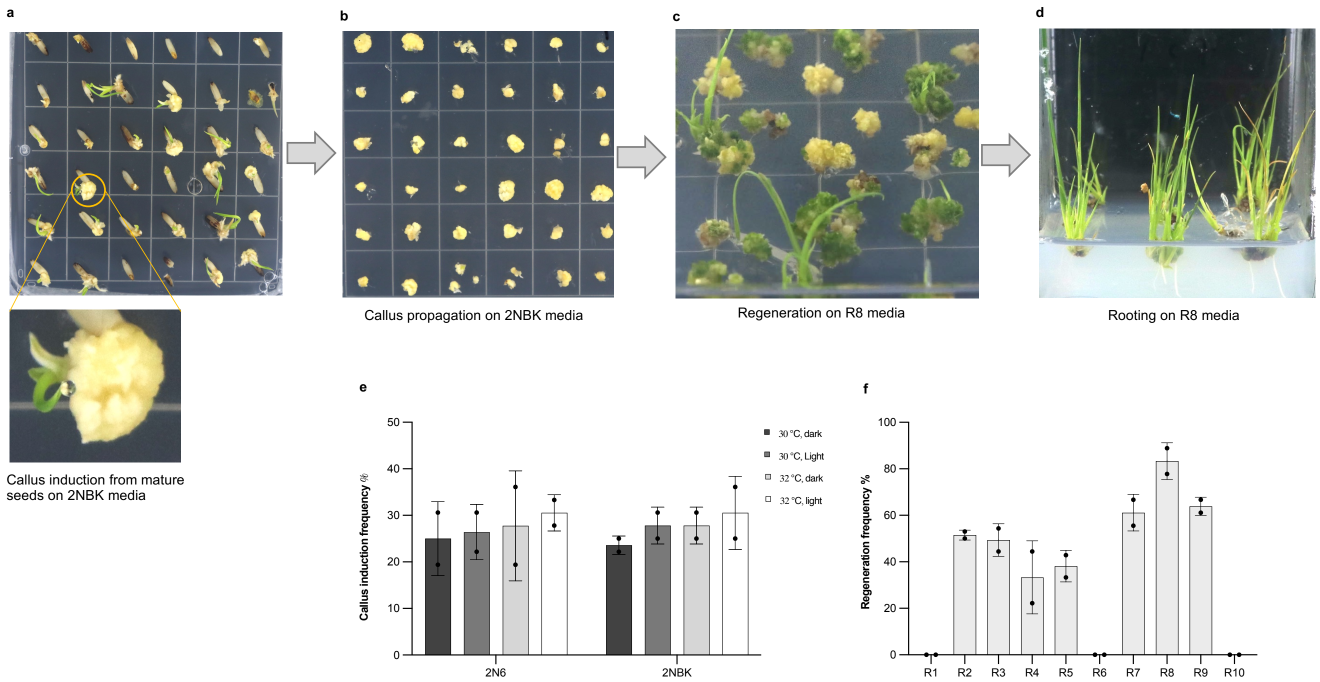

Supplementary Fig. 7. Main steps of the establishment of a regeneration protocol for Cempo Ireng. a) Callus was induced from mature seeds on 2NBK medium. b) Callus propagation on fresh 2NBK medium. c) Shoot induction using R8 medium. d) Rooting on MSRO medium. e) Callus induction frequency from rice grains using 2N6 and 2NBK media under 30 °C in the dark, 30 °C in the light, 32 °C in the dark, or 32 °C in light. Data show the percentage mean values ± 95% confidence interval of two independent experiments (*n* = 36). f) Regeneration frequency of Cempo Ireng on 10 different regeneration media recipes (R1–R10) was calculated and represented as percent mean values ± SE of regenerated shoots from 585 callus in two independent experiments.

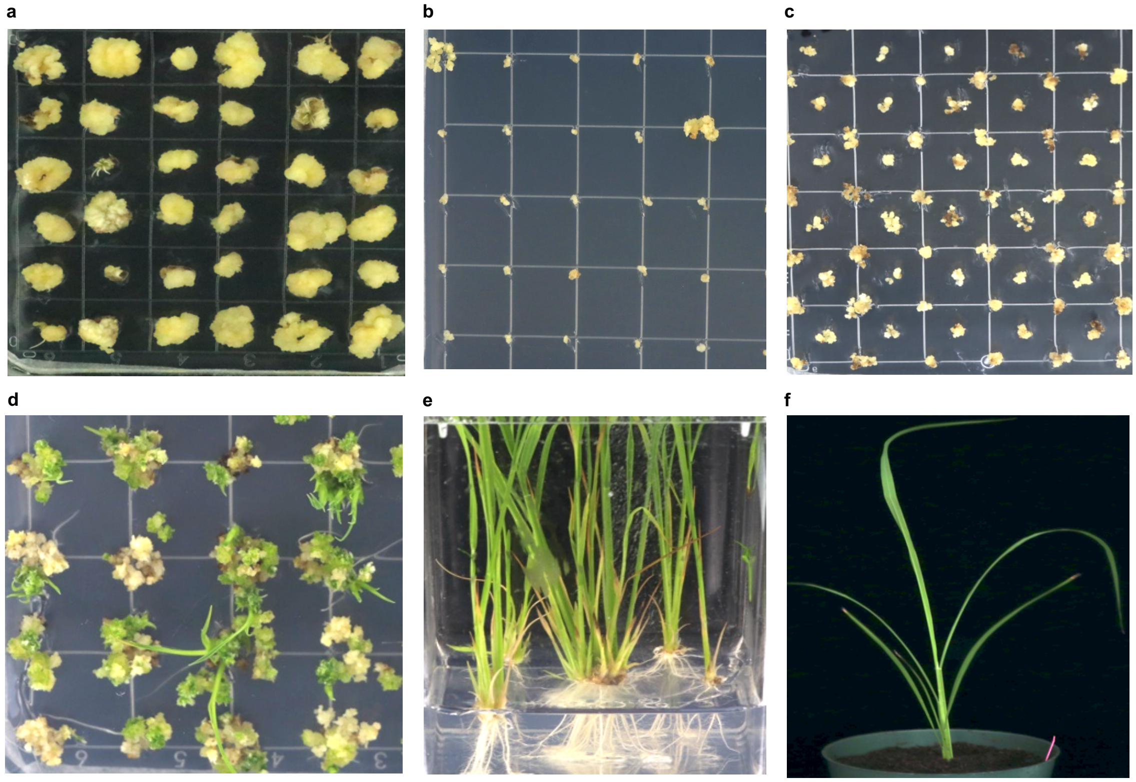

Supplementary Fig. 8. Different stages of the *Agrobacterium*-mediated transformation of Cempo Ireng. a) Embryogenic-like callus induction on callus-induction medium. b) First selection of transformed calli after co-cultivation on NBKCH20 medium containing 20 mg/L hygromycin and 200 mg/L timentin. c) Second selection of the surviving calli on the nNBKCH40 medium containing 40 mg/L hygromycin and 200 mg/L timentin. d) Shoot regeneration from antibiotic-resistant putative transformant calli on R8 regeneration medium containing 5 mg/L hygromycin. e) Rooting of putative transformed shootlets on ½ MS medium containing 5 mg/L hygromycin. f) Soil acclimatization of the plantlets in the greenhouse condition.

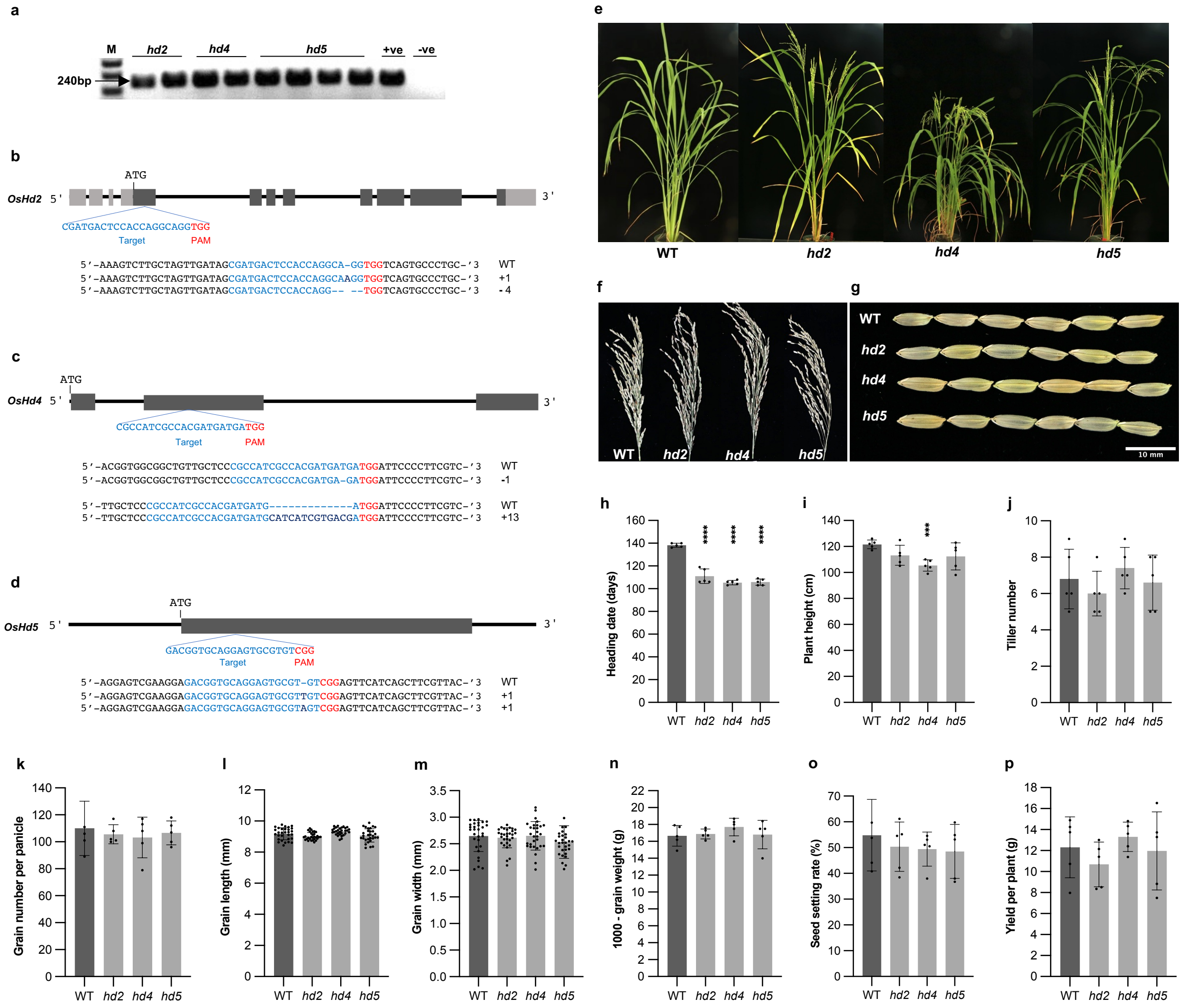

Supplementary Fig. 9. CRISPR/Cas9-induced knockout of three repressors of heading date in rice. a) Gel electrophoresis of the PCR products (240 bp) amplified from the T-DNA sequence in the engineered rice plants (*hd2, hd4, hd5*). b–d) Schematic map of the *Hd2* (b), *Hd4* (c), and *Hd5* (d) genomic loci and sgRNA target sites in Cempo Ireng. The PAM (NGG) is shown in red. A sequence alignment of the target region in the wild-type (WT) and mutant T1 lines shows the different insertion/deletions in the mutant lines. e) Representative image taken at the time of flowering in the mutant lines (*hd2*, *hd4*, and *hd5*), although the WT was not yet flowering. f) Representative image showing the morphology of the main panicles of the WT and mutant lines. g) Grain phenotypes of the mutant lines and WT, scale bar, 10 mm. h–p) Unpaired *t*-test comparing the heading-and yield-related traits of the mutant lines (each n=5) and WT (n=5). Data are presented as mean values with the error bars denoting 95% confidence intervals. Asterisks indicate significant differences to the WT: *, $p<0.05$; ***, $p<0.001$; ****, $p<0.0001$. h) Heading date. i) Plant height. j) Tiller number. k) Average grain number per panicle. l) Grain length, (n=30). m) Grain width, (n=30). n) Thousand-grain weight. o) Seed setting rate. p) Yield per plant.

a

```
Hd2-WT   MMGTAHHNQTAGSALGVGVGDANDAVPGAGGGGYSDPDGGPTSGVQPPPQVCWERFIQKKTIKVLLVESDDSTRQVV
Hd2-50.2 MMGTAHHNQTAGSALGVGVGDANDAVPGAGGGGYSDPDGGPTSGVQPPPQVCWERFIQKKTIKVLLVESDDSTRQGG

Hd2-WT   SALLRHCMYEVIPAENGQQAWTYLEDMQNSIDLVLTEVVMPGVSGISLLSRIMNHNICKNIPVIMMSSNDAMGTVFK
Hd2-50.2 QCPASSLHV*

Hd2-WT   CLSKGAVDFLVKPIRKNELKNLWQHVWRRCHSSSGSGSESGIQTQKCAKSKSGDESNNNSGSNDDDDDDGVIMGLNA
Hd2-50.2

Hd2-WT   RDGSDNGSGTQAQSSWTKRAVEIDSPQAMSPDQLADPPDSTCAQVIHLKSDICSNRWLPCTSNKNSKKQKETNDDFK
Hd2-50.2

Hd2-WT   GKDLEIGSPRNLNTAYQSSPNERSIKPTDRRNEYPLQNNSKEAAMENLEESSVRAADLIGSMAKNMDAQQAARATNA
Hd2-50.2

Hd2-WT   PNCSSKVPEGKDKNRDNIMPSLELSLKRSRSTGDGANAIQEEQRNVLRRSDLSAFTRYHTPVASNQGGTGFVGSCSP
Hd2-50.2

Hd2-WT   HDNSSEAMKTDSAYNMKSNSDAAPIKQGSNGSSNNNDMGSTTKNVVTKPSTNKERVMSPSAVKANGHTSAFHPAQHW
Hd2-50.2

Hd2-WT   TSPANTTGKEKTDEVANNAAKRAQPGEVQSNLVQHPRPILHYVNFDVSRENGGSGAPQCGSSNVFDPPVEGHAANYG
Hd2-50.2

Hd2-WT   VNGSNSGSNNGSNGQNGSTTAVNAERPNMEIANGTINKSGPGGGNGSGSGSGNDMYLKRFTQREHRVAAVIKFRQKR
Hd2-50.2

Hd2-WT   KERNFGKKVRYQSRKRLAEQRPRVRGQFVRQAVQDQQQQGGGREAAADR*
Hd2-50.2
```

b

```
Hd4-WT   MGMANEESPNYQVKKGGRIPPPRSSLIYPFMSMGPAAGEGCGLCGADGGGCCSRHRHDDDGFPFVFPPSACQGIGAP
Hd4-67.4 MGMANEESPNYQVKKGGRIPPPRSSLIYPFMSMGPAAGEGCGLCGADGGGCCSRHRHDDASS*

Hd4-WT   APPVHEFQFFGNDGGGDDGESVAWLFDDYPPPSPVAAAAGMHHRQPPYDGVVAPPSLFRRNTGAGGLTFDVSLGGRP
Hd4-67.4

Hd4-WT   DLDAGLGLGGGSGRHAEAAASATIMSYCGSTFTDAASSMPKEMVAAMADVGESLNPNTVVGAMVEREAKLMRYKEKR
Hd4-67.4

Hd4-WT   SLNPNTVVGAMVEREAKLMRYKEKRKKRCYEKQIRYASRKAYAEMRPRVRGRFAKEADQEAVAPPSTYVDPSRLELG
Hd4-67.4

Hd4-WT   QWFR*
Hd4-67.4
```

c

```
Hd5-WT    MKSRKSYGHLLSPVGSPPSDNESGAAAAAAAAGGGGCGSSAGYVVYGGGGGGDSPAKEQDRFLPIANVSRIMKRSLP
Hd5-81.3  MKSRKSYGHLLSPVGSPPSDNESGAAAAAAAAGGGGCGSSAGYVVYGGGGGGDSPAKEQDRFLPIANVSRIMKRSLP

Hd5-WT    ANAKISKEAKETVQECVSEFISFVTGEASDKCQREKRKTINGDDLLWAMTTLGFEAYVGPLKSYLNRYREAEGEKAA
Hd5-81.3  ANAKISKEAKETVQECVVGVHQLRYRRGLRQVPAREAEDHQRRRPPLGHDHAGVRGLRRPAQVLPQPLPRGRGREGR

Hd5-WT    VLGGAGGAAAARHGEGGCCGGGGGADGVVIDGHSPLAGGLSHSHHGHQQQDGGGDVGLMMGGGAAGVGYNAGAGSTT
Hd5-81.3  RARRRRRRRRGAPRRGRLLRRRRRRRWRRHRRAFPARRRPVTLTPWSSAAGRRRRRRAHDGWRRRRRRVQRRGRVDD

Hd5-WT    TAFYAPAATAASGNKAYCGGDGSRVMEFEGIGGEEESGGGGGGGERGFAGHLHGVQWFRLKRSTN*
Hd5-81.3  DGVLRAGGDGGVREQGVLRRRRVEGDGVRGHRRRGGERRRRRRRREGVRRPPPWRAMV*
```

Supplementary Fig. 10. Protein sequence alignment of the CRISPR mutants and the wild type (Cempo Ireng). Examples of the *hd2, hd4*, and *hd5* mutants with deletions or insertions. a) *hd2-50.2* has one nucleotide insertion. b) *hd4-67.4* has 13 nucleotide insertions. c) *hd5-81.3* has one nucleotide insertion. These indels changed the downstream amino acid sequence and created a premature stop codon in all mutant lines. Protein sequence upstream of the cut site is highlighted with yellow.

Supplementary Table 7. **Genotyping of the T0 and T1 edited plants.** The mutant lines are listed. Table shows the targeted gene, mutant generation, plant ID, the genotype detected compared to the wild-type sequence and the mutation type. sgRNA, PAM, and insertion sequences are written with light blue, red, and orange respectively.

Target	Generation	Plant ID	Genotype		Mutation
		WT	AGTCTTGCTAGTTGAGAGCGATGACTCCACCAGGCA-GGTGGTCA	WT	
	T0	*hd2-50*	AGTCTTGCTAGTTGAGAGCGATGACTCCACCAGGCAAGGTGGTCA<br>AGTCTTGCTAGTTGAGAGCGATGACTCCACCAGG-- --TGGTCA	+1<br>-4	Biallelic
	T0	*hd2-51*	AGTCTTGCTAGTTGAGAGCGATGACTCCACCAGGCAAGGTGGTCA<br>AGTCTTGCTAGTTGAGAGCGATGACTCCACCAGG-- --TGGTCA	+1<br>-4	Biallelic
	T1	*hd2-50.2*	AGTCTTGCTAGTTGAGAGCGATGACTCCACCAGGCAAGGTGGTCA<br>AGTCTTGCTAGTTGAGAGCGATGACTCCACCAGGCAAGGTGGTCA	+1<br>+1	Homozygous
**Hd2**	T1	*hd2-50.5*	AGTCTTGCTAGTTGAGAGCGATGACTCCACCAGG-- --TGGTCA<br>AGTCTTGCTAGTTGAGAGCGATGACTCCACCAGG-- --TGGTCA	-4<br>-4	Homozygous
	T1	*hd2-50.9*	AGTCTTGCTAGTTGAGAGCGATGACTCCACCAGG-- --TGGTCA<br>AGTCTTGCTAGTTGAGAGCGATGACTCCACCAGG-- --TGGTCA	-4<br>-4	Homozygous
	T1	*hd2-50.14*	AGTCTTGCTAGTTGAGAGCGATGACTCCACCAGG-- --TGGTCA<br>AGTCTTGCTAGTTGAGAGCGATGACTCCACCAGG-- --TGGTCA	-4<br>-4	Homozygous
	T1	*hd2-51.3*	AGTCTTGCTAGTTGAGAGCGATGACTCCACCAGGCAAGGTGGTCA<br>AGTCTTGCTAGTTGAGAGCGATGACTCCACCAGGCAAGGTGGTCA	+1<br>+1	Homozygous
	T1	*hd2-51.5*	AGTCTTGCTAGTTGAGAGCGATGACTCCACCAGG-- --TGGTCA<br>AGTCTTGCTAGTTGAGAGCGATGACTCCACCAGG-- --TGGTCA	-4<br>-4	Homozygous
		WT	GGTGGCGGCTGTTGCTCCCGCCATCGCCACGATGATGATGGATTC	WT	
	T0	*hd4-67*	GGTGGCGGCTGTTGCTCCCGCCATCGCCACGATGA-GATGGATTC<br>GTTGCTCCCGCCATCGCCACGATGCATCATCGTGACGATGGATTC	-1<br>+13	Biallelic
	T0	*hd4-68*	GGTGGCGGCTGTTGCTCCCGCCATCGCCACGATGA-GATGGATTC<br>GTTGCTCCCGCCATCGCCACGATGCATCATCGTGACGATGGATTC	-1<br>+13	Biallelic
	T0	*hd4-69*	GGTGGCGGCTGTTGCTCCCGCCATCGCCACGATGA-GATGGATTC<br>GTTGCTCCCGCCATCGCCACGATGCATCATCGTGACGATGGATTC	-1<br>+13	Biallelic
**Hd4**	T1	*hd4-67.1*	GTTGCTCCCGCCATCGCCACGATGCATCATCGTGACGATGGATTC<br>GTTGCTCCCGCCATCGCCACGATGCATCATCGTGACGATGGATTC	+13<br>+13	Homozygous
	T1	*hd4-67.2*	GGTGGCGGCTGTTGCTCCCGCCATCGCCACGATGA-GATGGATTC<br>GGTGGCGGCTGTTGCTCCCGCCATCGCCACGATGA-GATGGATTC	-1<br>-1	Homozygous
	T1	*hd4-67.4*	GTTGCTCCCGCCATCGCCACGATGCATCATCGTGACGATGGATTC<br>GTTGCTCCCGCCATCGCCACGATGCATCATCGTGACGATGGATTC	+13<br>+13	Homozygous
	T1	*hd4-68.3*	GGTGGCGGCTGTTGCTCCCGCCATCGCCACGATGA-GATGGATTC<br>GGTGGCGGCTGTTGCTCCCGCCATCGCCACGATGA-GATGGATTC	-1<br>-1	Homozygous
	T1	*hd4-68.7*	GGTGGCGGCTGTTGCTCCCGCCATCGCCACGATGA-GATGGATTC<br>GGTGGCGGCTGTTGCTCCCGCCATCGCCACGATGA-GATGGATTC	-1<br>-1	Homozygous
		WT	CTCCAAGGAGTCGAAGGAGACGGTGCAGGAGTGCG-TGTCGGAGT	WT	
	T0	*hd5-1*	CTCCAAGGAGTCGAAGGAGACGGTGCAGGAGTGCGATGTCGGAGT<br>CTCCAAGGAGTCGAAGGAGACGGTGCAGGAGTGCGTTGTCGGAGT	+1<br>+1	Biallelic
	T0	*hd5-2*	CTCCAAGGAGTCGAAGGAGACGGTGCAGGAGTGCGTTGTCGGAGT<br>CTCCAAGGAGTCGAAGGAGACGGTGCAGGAGTGCGTTGTCGGAGT	+1<br>+1	Homozygous
	T0	*hd5-5*	CTCCAAGGAGTCGAAGGAGACGGTGCAGGAGTGCG-TGTCGGAGT<br>CTCCAAGGAGTCGAAGGAGACGGTGCAGGAGTGCGTTGTCGGAGT	0<br>+1	Heterozygous
	T0	*hd5-6*	CTCCAAGGAGTCGAAGGAGACGGTGCAGGAGTGCG-TGTCGGAGT<br>CTCCAAGGAGTCGAAGGAGACGGTGCAGGAGTGCGTTGTCGGAGT<br>GTGCAGGAGTGCAGCGCACAGGAGTGCGACCGAG---GTCGGAGT	0<br>+1<br>-2,+30	Chimeric
	T0	*hd5-7*	CTCCAAGGAGTCGAAGGAGACGGTGCAGGAGTGCG-TGTCGGAGT<br>CTCCAAGGAGTCGAAGGAGACGGTGCAGGAGTGCGTTGTCGGAGT<br>CTCCAAGGAGTCGAAGGAGACGGTGCAGGAGTGCGATGTCGGAGT	0<br>+1<br>+1	Chimeric
	T0	*hd5-8*	CTCCAAGGAGTCGAAGGAGACGGTGCAGGAGTGCGATGTCGGAGT<br>CTCCAAGGAGTCGAAGGAGACGGTGCAGGAGTGCGATGTCGGAGT	+1<br>+1	Homozygous
	T0	*hd5-9*	CTCCAAGGAGTCGAAGGAGACGGTGCAGGAGTGCGATGTCGGAGT<br>CTCCAAGGAGTCGAAGGAGACGGTGCAGGAGTGCGTTGTCGGAGT	+1<br>+1	Biallelic

Hd5	T0	*hd5-24*	CTCCAAGGAGTCGAAGGAGACGGTGCAGGAGTGCGATGTCGGAGT<br>CTCCAAGGAGTCGAAGGAGACGGTGCAGGAGTGCGATGTCGGAGT	+1<br>+1	Homozygous
	T0	*hd5-25*	CTCCAAGGAGTCGAAGGAGACGGTGCAGGAGTGCGCTGTCGGAGT<br>CTCCAAGGAGTCGAAGGAGACGGTGCAGGAGTGCGTTGTCGGAGT	+1<br>+1	Biallelic
	T0	*hd5-44*	CTCCAAGGAGTCGAAGGAGACGGTGCAGGAGTGCG-TGTCGGAGT<br>CTCCAAGGAGTCGAAGGAGACGGTGCAGGAGTGCGTTGTCGGAGT<br>CTCCAAGGAGTCGAAGGAGACGGTGCAGGAGTGCGATGTCGGAGT	0<br>+1<br>+1	Chimeric
	T0	*hd5-45*	CTCCAAGGAGTCGAAGGAGACGGTGCAGGAGTGCGATGTCGGAGT<br>CTCCAAGGAGTCGAAGGAGACGGTGCAGGAGTGCGTTGTCGGAGT	+1<br>+1	Biallelic
	T0	*hd5-46*	CTCCAAGGAGTCGAAGGAGACGGTGCAGGAGTGCGATGTCGGAGT<br>CTCCAAGGAGTCGAAGGAGACGGTGCAGGAGTGCGTTGTCGGAGT	+1<br>+1	Biallelic
	T0	*hd5-79*	CTCCAAGGAGTCGAAGGAGACGGTGCAGGAGTGCGTTGTCGGAGT<br>CTCCAAGGAGTCGAAGGAGACGGTGCAGGAGTGCGTTGTCGGAGT	+1<br>+1	Homozygous
	T0	*hd5-80*	CTCCAAGGAGTCGAAGGAGACGGTGCAGGAGTGCGATGTCGGAGT<br>CTCCAAGGAGTCGAAGGAGACGGTGCAGGAGTGCGATGTCGGAGT	+1<br>+1	Homozygous
	T0	*hd5-81*	CTCCAAGGAGTCGAAGGAGACGGTGCAGGAGTGCGTTGTCGGAGT<br>CTCCAAGGAGTCGAAGGAGACGGTGCAGGAGTGCGTTGTCGGAGT	+1<br>+1	Homozygous
	T0	*hd5-82*	CTCCAAGGAGTCGAAGGAGACGGTGCAGGAGTGCGATGTCGGAGT<br>CTCCAAGGAGTCGAAGGAGACGGTGCAGGAGTGCGATGTCGGAGT	+1<br>+1	Homozygous
	T0	*hd5-83*	CTCCAAGGAGTCGAAGGAGACGGTGCAGGAGTGCGTTGTCGGAGT<br>CTCCAAGGAGTCGAAGGAGACGGTGCAGGAGTGCGTTGTCGGAGT	+1<br>+1	Homozygous
	T0	*hd5-84*	CTCCAAGGAGTCGAAGGAGACGGTGCAGGAGTGCG-TGTCGGAGT<br>CTCCAAGGAGTCGAAGGAGACGGTGCAGGAGTGCGCTGTCGGAGT<br>CTCCAAGGAGTCGAAGGAGACGGTGCAGGAGTGCGATGTCGGAGT	0<br>+1<br>+1	Chimeric
	T0	*hd5-85*	CTCCAAGGAGTCGAAGGAGACGGTGCAGGAGTGCGATGTCGGAGT<br>CTCCAAGGAGTCGAAGGAGACGGTGCAGGAGTGCGTTGTCGGAGT	+1<br>+1	Biallelic
	T0	*hd5-86*	CTCCAAGGAGTCGAAGGAGACGGTGCAGGAGTGCG-TGTCGGAGT<br>CTCCAAGGAGTCGAAGGAGACGGTGCAGGAGTGCGTTGTCGGAGT	0<br>+1	Heterozygous
	T0	*hd5-87*	CTCCAAGGAGTCGAAGGAGACGGTGCAGGAGTGCGATGTCGGAGT<br>CTCCAAGGAGTCGAAGGAGACGGTGCAGGAGTGCGTTGTCGGAGT	+1<br>+1	Biallelic
	T0	*hd5-92*	CTCCAAGGAGTCGAAGGAGACGGTGCAGGAGTGCG-TGTCGGAGT<br>CTCCAAGGAGTCGAAGGAGACGGTGCAGGAGTGCGTTGTCGGAGT	0<br>+1	Heterozygous
	T0	*hd5-93*	CTCCAAGGAGTCGAAGGAGACGGTGCAGGAGTGCGATGTCGGAGT<br>CTCCAAGGAGTCGAAGGAGACGGTGCAGGAGTGCGGTGTCGGAGT	+1<br>+1	Biallelic
	T0	*hd5-94*	CTCCAAGGAGTCGAAGGAGACGGTGCAGGAGTGCGATGTCGGAGT<br>CTCCAAGGAGTCGAAGGAGACGGTGCAGGAGTGCGGTGTCGGAGT	+1<br>+1	Biallelic
	T0	*hd5-95*	CTCCAAGGAGTCGAAGGAGACGGTGCAGGAGTGCGATGTCGGAGT<br>CTCCAAGGAGTCGAAGGAGACGGTGCAGGAGTGCGGTGTCGGAGT	+1<br>+1	Biallelic
	T0	*hd5-96*	CTCCAAGGAGTCGAAGGAGACGGTGCAGGAGTGCG-TGTCGGAGT<br>CTCCAAGGAGTCGAAGGAGACGGTGCAGGAGTGCGTTGTCGGAGT	0<br>+1	Heterozygous
	T0	*hd5-97*	CTCCAAGGAGTCGAAGGAGACGGTGCAGGAGTGCG-TGTCGGAGT<br>CTCCAAGGAGTCGAAGGAGACGGTGCAGGAGTGCGATGTCGGAGT	0<br>+1	Heterozygous
	T0	*hd5-98*	CTCCAAGGAGTCGAAGGAGACGGTGCAGGAGTGCG-TGTCGGAGT<br>CTCCAAGGAGTCGAAGGAGACGGTGCAGGAGTGCGATGTCGGAGT	0<br>+1	Heterozygous
	T0	*hd5-99*	CTCCAAGGAGTCGAAGGAGACGGTGCAGGAGTGCG-TGTCGGAGT<br>CTCCAAGGAGTCGAAGGAGACGGTGCAGGAGTGCGTTGTCGGAGT	0<br>+1	Heterozygous
	T0	*hd5-100*	CTCCAAGGAGTCGAAGGAGACGGTGCAGGAGTGCGTTGTCGGAGT<br>CTCCAAGGAGTCGAAGGAGACGGTGCAGGAGTGCGTTGTCGGAGT	+1<br>+1	Homozygous
	T0	*hd5-102*	CTCCAAGGAGTCGAAGGAGACGGTGCAGGAGTGCG-TGTCGGAGT<br>CTCCAAGGAGTCGAAGGAGACGGTGCAGGAGTGCGATGTCGGAGT	0<br>+1	Heterozygous
	T1	*hd5-2.2*	CTCCAAGGAGTCGAAGGAGACGGTGCAGGAGTGCGTTGTCGGAGT<br>CTCCAAGGAGTCGAAGGAGACGGTGCAGGAGTGCGTTGTCGGAGT	+1<br>+1	Homozygous
	T1	*hd5-2.3*	CTCCAAGGAGTCGAAGGAGACGGTGCAGGAGTGCGTTGTCGGAGT<br>CTCCAAGGAGTCGAAGGAGACGGTGCAGGAGTGCGTTGTCGGAGT	+1<br>+1	Homozygous
	T1	*hd5-2.8*	CTCCAAGGAGTCGAAGGAGACGGTGCAGGAGTGCGTTGTCGGAGT<br>CTCCAAGGAGTCGAAGGAGACGGTGCAGGAGTGCGTTGTCGGAGT	+1<br>+1	Homozygous
	T1	*hd5-2.9*	CTCCAAGGAGTCGAAGGAGACGGTGCAGGAGTGCGTTGTCGGAGT<br>CTCCAAGGAGTCGAAGGAGACGGTGCAGGAGTGCGTTGTCGGAGT	+1<br>+1	Homozygous
	T1	*hd5-8.1*	CTCCAAGGAGTCGAAGGAGACGGTGCAGGAGTGCGATGTCGGAGT<br>CTCCAAGGAGTCGAAGGAGACGGTGCAGGAGTGCGATGTCGGAGT	+1<br>+1	Homozygous

T1	*hd5-8.2*	CTCCAAGGAGTCGAAGGAGACGGTGCAGGAGTGCGATGTCGGAGT<br>CTCCAAGGAGTCGAAGGAGACGGTGCAGGAGTGCGATGTCGGAGT	+1<br>+1	Homozygous
T1	*hd5-8.13*	CTCCAAGGAGTCGAAGGAGACGGTGCAGGAGTGCGATGTCGGAGT<br>CTCCAAGGAGTCGAAGGAGACGGTGCAGGAGTGCGATGTCGGAGT	+1<br>+1	Homozygous
T1	*hd5-79.6*	CTCCAAGGAGTCGAAGGAGACGGTGCAGGAGTGCGTTGTCGGAGT<br>CTCCAAGGAGTCGAAGGAGACGGTGCAGGAGTGCGTTGTCGGAGT	+1<br>+1	Homozygous
T1	*hd5-79.10*	CTCCAAGGAGTCGAAGGAGACGGTGCAGGAGTGCGTTGTCGGAGT<br>CTCCAAGGAGTCGAAGGAGACGGTGCAGGAGTGCGTTGTCGGAGT	+1<br>+1	Homozygous
T1	*hd5-79.17*	CTCCAAGGAGTCGAAGGAGACGGTGCAGGAGTGCGTTGTCGGAGT<br>CTCCAAGGAGTCGAAGGAGACGGTGCAGGAGTGCGTTGTCGGAGT	+1<br>+1	Homozygous
T1	*hd5-80.11*	CTCCAAGGAGTCGAAGGAGACGGTGCAGGAGTGCGATGTCGGAGT<br>CTCCAAGGAGTCGAAGGAGACGGTGCAGGAGTGCGATGTCGGAGT	+1<br>+1	Homozygous
T1	*hd5-80.12*	CTCCAAGGAGTCGAAGGAGACGGTGCAGGAGTGCGATGTCGGAGT<br>CTCCAAGGAGTCGAAGGAGACGGTGCAGGAGTGCGATGTCGGAGT	+1<br>+1	Homozygous
T1	*hd5-81.3*	CTCCAAGGAGTCGAAGGAGACGGTGCAGGAGTGCGTTGTCGGAGT<br>CTCCAAGGAGTCGAAGGAGACGGTGCAGGAGTGCGTTGTCGGAGT	+1<br>+1	Homozygous
T1	*hd5-81.4*	CTCCAAGGAGTCGAAGGAGACGGTGCAGGAGTGCGTTGTCGGAGT<br>CTCCAAGGAGTCGAAGGAGACGGTGCAGGAGTGCGTTGTCGGAGT	+1<br>+1	Homozygous
T1	*hd5-82.1*	CTCCAAGGAGTCGAAGGAGACGGTGCAGGAGTGCGATGTCGGAGT<br>CTCCAAGGAGTCGAAGGAGACGGTGCAGGAGTGCGATGTCGGAGT	+1<br>+1	Homozygous
T1	*hd5-82.2*	CTCCAAGGAGTCGAAGGAGACGGTGCAGGAGTGCGATGTCGGAGT<br>CTCCAAGGAGTCGAAGGAGACGGTGCAGGAGTGCGATGTCGGAGT	+1<br>+1	Homozygous
T1	*hd5-82.5*	CTCCAAGGAGTCGAAGGAGACGGTGCAGGAGTGCGATGTCGGAGT<br>CTCCAAGGAGTCGAAGGAGACGGTGCAGGAGTGCGATGTCGGAGT	+1<br>+1	Homozygous

Supplementary Table 8. List of oligos. Table lists the oligo sequences used for designing the sgRNAs for the targeted genes and the primers used for detecting the T-DNA insertion and mutant genotypes. Blue nucleotides represent the BsaI restriction overhang.

Name	Sequence	Usage
Hd2-sgRNA1-F	GGCACGATGACTCCACCAGGCAGG	Design Hd2 sgRNA
Hd2-sgRNA1-R	AAACCCTGCCTGGTGGAGTCATCG	Design Hd2 sgRNA
Hd4-sgRNA1-F	GGCACGCCATCGCCACGATGATGA	Design Hd4 sgRNA
Hd4-sgRNA1-R	AAACTCATCATCGTGGCGATGGCG	Design Hd4 sgRNA
Hd5-sgRNA1-F	GGCAGACGGTGCAGGAGTGCGTGT	Design Hd5 sgRNA
Hd5-sgRNA1-R	AAACACACGCACTCCTGCACCGTC	Design Hd5 sgRNA
Hd2-F1	ATGATGGGAACCGCTCATCAC	Amplify Hd2 target region
Hd2-R6	CATACATGCAGTGACGAAGC	Amplify Hd2 target region
Hd4-F2	ATGTCGATGGGACCAGCAGC	Amplify Hd4 target region
Hd4-R3	GATGGTGGCGCTGGCCGCGG	Amplify Hd4 target region
Hd5-F2	ACTTGCTGAGCCCGGTGGGC	Amplify Hd5 target region
Hd5-R3	GCGTGGTCATGGCCCAGAGG	Amplify Hd5 target region
Cas9-F7	GATCGACCTGTCTCAGCTGG	Amplify 240 bp of the T-DNA sequence
Nos-R7	CGGCAACAGGATTCAATCTTAAG	Amplify 240 bp of the T-DNA sequence
T7-F	TAA TAC GAC TCA CTA TAG GG	Sequence the cloned DNA into pjet vector

Supplementary File 1. Functional annotation of the predicted genes. Gene annotation was performed according to the best match for each predicted protein against the NR NCBI protein database using Diamond Blastp. Columns A, C, and E list the number of genes involved in the three main Gene ontology (GO) terms (biological process (column B), molecular function (column D), and cellular component (column F) are listed for the five genome sequences (Cempo Ireng, Pulut Hitam-2, Balatinaw, Cempo Abang, and Zag). A separate spreadsheet was made for each variety.

Supplementary File 2. Repetitive sequences in the genome of pigmented rice. The Repetitive sequence distribution in the five sequenced genomes (Cempo Ireng, Pulut Hitam-2, Balatinaw, Cempo Abang, and Zag) are listed. Columns A and B show the ten main transposable elements classes and subclasses. Column C-E shows the number of the identified repetitive sequences, their size, and percentage in the genome. A separate spreadsheet was made for each variety.

Supplementary File 3. Metabolic profiling of pigmented rice. Columns A and B list the nine super-pathway and 59 sub-pathways, respectively. Column C lists the identified 625 biochemicals (575 known identity, 50 unknown). Column D-BN shows the average expression of the identified biochemical relative to the median value of these chemicals in all pigmented rice accessions.

Supplementary File 4. Metal ion profiling of pigmented rice. The file shows the quantitative measurement of 22 metal ions in 63 pigmented accessions (24 black, 35 red, and 4 brown). Columns A and B list the metal ion name and abbreviation, respectively. The measurements were calculated in the microgram scale relative to the 1 gram of grain weight as shown in column C. Column D-BN shows the metal content (μg) in all pigmented rice accessions.

Supplementary File 5. Correlation analysis between grain pigments and metal ion concentration. The file shows the statistical correlation between the main grain pigments (anthocyanin and proanthocyanidins) and the 22 analyzed metal ion concentrations. Pearson's correlation coefficients (R) and P-values (P) were calculated. The correlation was considered to be significant at the 0.05 level.

References

1. Martin, M. Cutadapt removes adapter sequences from high-throughput sequencing reads. *EMBnet J.* **17**, 3 (2011).
2. Bolger, A.M., Lohse, M. & Usadel, B. Trimmomatic: a flexible trimmer for Illumina sequence data. *Bioinformatics* **30**, 2114-2120 (2014).
3. Li, H. & Durbin, R. Fast and accurate long-read alignment with Burrows–Wheeler transform. *Bioinformatics* **26**, 589-595 (2010).
4. Danecek, P. et al. Twelve years of SAMtools and BCFtools. *GigaScience* **10** (2021).
5. McKenna, A. et al. The genome analysis toolkit: a MapReduce framework for analyzing next-generation DNA sequencing data. *Genome Res.* **20**, 1297-1303 (2010).
6. Van der Auwera, G.A. et al. From fastQ data to high-confidence variant calls: the genome analysis toolkit best practices pipeline. *Curr. Protoc. Bioinformatics* **43**, 11.10.11-11.10.33 (2013).
7. Wang, W. et al. Genomic variation in 3,010 diverse accessions of Asian cultivated rice. *Nature* **557**, 43-49 (2018).
8. Zhou, Y. et al. A platinum standard pan-genome resource that represents the population structure of Asian rice. *Scientific Data* **7**, 113 (2020).
9. Danecek, P. et al. The variant call format and VCFtools. *Bioinformatics (Oxford, England)* **27**, 2156-2158 (2011).
10. Purcell, S. et al. PLINK: a tool set for whole-genome association and population-based linkage analyses. *Am. J. Hum. Genet.* **81**, 559-575 (2007).
11. Hiei, Y. & Komari, T. Agrobacterium-mediated transformation of rice using immature embryos or calli induced from mature seed. *Nat. Protoc.* **3**, 824-834 (2008).
