# Supplementary material for: Multi-omics resources for targeted agronomic improvement of pigmented rice
Source: Nat Food. 2023 May 11;4(5):366–71. doi: 10.1038/s43016-023-00742-9 (PMC10208962; doi:10.1038/s43016-023-00742-9)
Supplement: Supplementary file 2 — Reporting Summary [file 43016_2023_742_MOESM2_ESM.pdf]

nature portfolio

Corresponding author(s): Magdy M. Mahfouz

Last updated by author(s): Mar 16, 2022

Reporting Summary

Nature Portfolio wishes to improve the reproducibility of the work that we publish. This form provides structure for consistency and transparency in reporting. For further information on Nature Portfolio policies, see our Editorial Policies and the Editorial Policy Checklist.

Statistics

For all statistical analyses, confirm that the following items are present in the figure legend, table legend, main text, or Methods section.

n/a	Confirmed	
☐	☒	The exact sample size (*n*) for each experimental group/condition, given as a discrete number and unit of measurement
☐	☒	A statement on whether measurements were taken from distinct samples or whether the same sample was measured repeatedly
☐	☒	The statistical test(s) used AND whether they are one- or two-sided<br>*Only common tests should be described solely by name; describe more complex techniques in the Methods section.*
☒	☐	A description of all covariates tested
☐	☒	A description of any assumptions or corrections, such as tests of normality and adjustment for multiple comparisons
☐	☒	A full description of the statistical parameters including central tendency (e.g. means) or other basic estimates (e.g. regression coefficient) AND variation (e.g. standard deviation) or associated estimates of uncertainty (e.g. confidence intervals)
☐	☒	For null hypothesis testing, the test statistic (e.g. *F*, *t*, *r*) with confidence intervals, effect sizes, degrees of freedom and *P* value noted<br>*Give P values as exact values whenever suitable.*
☒	☐	For Bayesian analysis, information on the choice of priors and Markov chain Monte Carlo settings
☒	☐	For hierarchical and complex designs, identification of the appropriate level for tests and full reporting of outcomes
☒	☐	Estimates of effect sizes (e.g. Cohen's *d*, Pearson's *r*), indicating how they were calculated

Our web collection on statistics for biologists contains articles on many of the points above.

Software and code

Policy information about availability of computer code

Data collection	All of the software used in this study is publicly available, and codes are available on request.
Data analysis	Prism files and Excel worksheets were used for analyzing data

For manuscripts utilizing custom algorithms or software that are central to the research but not yet described in published literature, software must be made available to editors and reviewers. We strongly encourage code deposition in a community repository (e.g. GitHub). See the Nature Portfolio guidelines for submitting code & software for further information.

Data

Policy information about availability of data

All manuscripts must include a data availability statement. This statement should provide the following information, where applicable:

- Accession codes, unique identifiers, or web links for publicly available datasets
- A description of any restrictions on data availability
- For clinical datasets or third party data, please ensure that the statement adheres to our policy

The data supporting our findings of this study are included within the article and supplementary information files. The raw genomics sequences and assemblies have been deposited to the National Center for Biotechnology Information under the BioProject accession code PRJNA942452. Mass spectrometry metabolomic data have been deposited to the MetaboLights database with the identifier number MTBLS3320.

Human research participants

Policy information about studies involving human research participants and Sex and Gender in Research.

Reporting on sex and gender	Our study does not include any human research participants.
Population characteristics	NA
Recruitment	NA
Ethics oversight	NA

Note that full information on the approval of the study protocol must also be provided in the manuscript.

Field-specific reporting

Please select the one below that is the best fit for your research. If you are not sure, read the appropriate sections before making your selection.

☒ Life sciences ☐ Behavioural & social sciences ☐ Ecological, evolutionary & environmental sciences

For a reference copy of the document with all sections, see nature.com/documents/nr-reporting-summary-flat.pdf

Life sciences study design

All studies must disclose on these points even when the disclosure is negative.

Sample size	For metabolome and ionome profiling, we used rice grains of 24 black, 35 red and four brown Asian rice. For genome sequencing, we collected DNA samples from the leaf of 51 individual plants (24 black, and 27 red).
Data exclusions	No data was excluded.
Replication	For metabolome and ionome profiling, 20 grains were grinded for each verity that were collected from a batch of grains collected from several individual plants, Three independent technical replicates were performed by Metabolon. For genome sequencing, a single seed per verity was grown for DNA collection.
Randomization	Grains were selected randomly and sample injection was also randomized in the UPLC–MS, and ICP–MS platforms. CRISPR-edited and wild type plants were selected in a random manner for harvesting and phenotyping.
Blinding	Blinding is not common practice for this type of experiments.

Reporting for specific materials, systems and methods

We require information from authors about some types of materials, experimental systems and methods used in many studies. Here, indicate whether each material, system or method listed is relevant to your study. If you are not sure if a list item applies to your research, read the appropriate section before selecting a response.

Materials & experimental systems

n/a	Involved in the study	
☒	☐	Antibodies
☒	☐	Eukaryotic cell lines
☒	☐	Palaeontology and archaeology
☒	☐	Animals and other organisms
☒	☐	Clinical data
☒	☐	Dual use research of concern

Methods

n/a	Involved in the study	
☒	☐	ChIP-seq
☒	☐	Flow cytometry
☒	☐	MRI-based neuroimaging
